# Tumor and α‐SMA‐expressing stromal cells in pancreatic neuroendocrine tumors have a distinct RNA profile depending on tumor grade

**DOI:** 10.1002/1878-0261.13727

**Published:** 2024-09-08

**Authors:** Helvijs Niedra, Raitis Peculis, Rihards Saksis, Ilona Mandrika, Sofija Vilisova, Jurijs Nazarovs, Austra Breiksa, Aija Gerina, Julie Earl, Ignacio Ruz‐Caracuel, Marta Gabriela Rosas, Aldis Pukitis, Natalja Senterjakova, Vita Rovite

**Affiliations:** ^1^ Department of Molecular and Functional Genomics Latvian Biomedical Research and Study Centre Riga Latvia; ^2^ Oncology clinic Pauls Stradins Clinical University Hospital Riga Latvia; ^3^ Institute of Pathology Pauls Stradins Clinical University Hospital Riga Latvia; ^4^ Department of Pathology Riga Stradins University Latvia; ^5^ Ramón y Cajal Health Research Institute (IRYCIS) Ramón y Cajal University Hospital. Ctra. Colmenar Viejo, CIBERONC Madrid Spain; ^6^ Department of Pathology Ramón y Cajal University Hospital. Ctra Colmenar Viejo Madrid Spain; ^7^ Centre of Gastroenterology, Hepatology and Nutrition Therapy Pauls Stradins Clinical University Hospital Riga Latvia

**Keywords:** digital spatial profiling, gene expression, Pancreatic neuroendocrine tumors, pathway enrichment analysis, tumor stroma

## Abstract

Alpha‐smooth muscle actin (α‐SMA) expression in the stroma is linked to the presence of cancer‐associated fibroblasts and is known to correlate with worse outcomes in various tumors. In this study, using a GeoMx digital spatial profiling approach, we characterized the gene expression of the tumor and α‐SMA‐expressing stromal cell compartments in pancreatic neuroendocrine tumors (PanNETs). The profiling was performed on tissues from eight retrospective cases (three grade 1, four grade 2, and one grade 3). Selected regions of interest were segmented geometrically based on tissue morphology and fluorescent signals from synaptophysin and α‐SMA markers. The α‐SMA‐expressing stromal‐cell‐associated genes were involved in pathways of extracellular matrix modification, whereas, in tumor cells, the gene expression profiles were associated with pathways involved in cell proliferation. The comparison of gene expression profiles across all three PanNET grades revealed that the differences between grades are not only present at the level of the tumor but also in the α‐SMA‐expressing stromal cells. Furthermore, the tumor cells from regions with a rich presence of adjacent α‐SMA‐expressing stromal cells revealed an upregulation of matrix metalloproteinase‐9 (*MMP9*) expression in grade 3 tumors. This study provides an in‐depth characterization of gene expression profiles in α‐SMA‐expressing stromal and tumor cells, and outlines potential crosstalk mechanisms.

## Graphical abstract

Alpha‐smooth muscle actin (α‐SMA) expression in the tumor stroma is linked to activation of cancer‐associated fibroblasts and is known to correlate with worse outcomes in various tumors. In this study, we aimed to profile tumor cells and α‐SMA stromal cells separately and identify their respective gene expression signatures across three pancreatic neuroendocrine tumor grades.

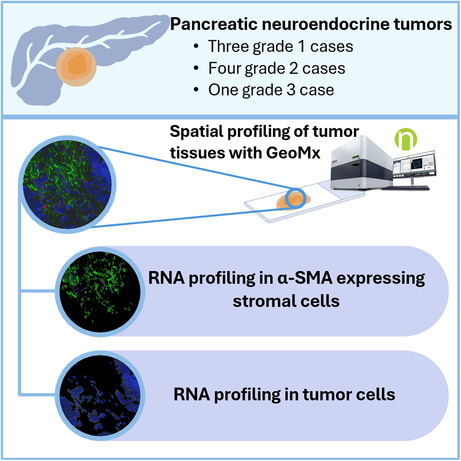

**Abbreviations**
AOI: area of illumination
ATRX: alternative lengthening of telomeres protein
CAF: cancer‐associated fibroblast
CD99: cluster of differentiation 99
COL1A1: type I collagen alpha chain 1
COL1A2: type I collagen alpha chain 2
COL3A1: type III collagen alpha chain 1
COL5A1: type V collagen alpha chain 1
COL5A2: type V collagen alpha chain 2
COL6A3: type VI collagen alpha chain 3
DAXX: death domain associated protein
DEG: differentially expressed gene
DKK: Dickkopf family proteins
DSP: digital spatial profiling
FDR: false discovery rate
FGF: fibroblast growth factor
FN1: fibronectin 1
G1/G2/G3: grade 1/grade 2/grade 3
JAG1: Jagged1
MMP9: matrix metallopeptidase 9
NET: neuroendocrine tumor
NOTCH3: neurogenic locus notch homolog protein 3
PanNET: pancreatic neuroendocrine tumor
PDGF: platelet‐derived growth factor
PDGFR: platelet‐derived growth factor receptor
PPI: protein–protein interaction
RBP4: retinol binding protein 4
RNA‐seq: ribonucleic acid sequencing
ROI: region of interest
TGF: tumor growth factor
TME: tumor microenvironment
VEGF: vascular endothelial growth factor
α‐SMA: alpha‐smooth muscle actin

## 1 Introduction

Neuroendocrine tumors (NETs) are a heterogeneous group of malignancies arising in the neuroendocrine system. The incidence of NETs is rising due to increased disease awareness and improved diagnostic accuracy. This trend and estimated prevalence (35 per 100 000) [1] highlight the importance of discovering novel biomarkers to enhance diagnostic accuracy and optimize disease management.

PanNETs make up about 7% of all NETs and less than 2% of all pancreatic neoplasms [2]. PanNETs arise from islets of Langerhans, specifically from α and β cells of the islets [3]. Based on clinical presentation, PanNETs are classified as non‐functioning PanNETs (50–90%) and functioning PanNETs (10–50%). Non‐functioning PanNET cells can still express specific pancreatic hormones. However, if the secretion levels are insufficient to cause hypersecretion‐related syndromes, they are classified as non‐functioning [4]. Thereby, non‐functioning PanNETs usually present with nonspecific symptoms such as abdominal pain, weight loss, or mass effect due to tumor or metastasis. Non‐functioning, low‐grade, and smaller (≤ 2 cm in diameter) PanNETs can display a more benign behavior, with slow growth and a better prognosis [5, 6].

Histologically, PanNETs are well‐differentiated tumors and, based on mitotic count and Ki‐67 index, they can be further classified into three grades: grade 1 (G1), grade 2 (G2), and grade 3 (G3) [7]. G1 and G2 tumors usually present hypervascular masses, with mitotic rate < 2 and Ki‐67 < 3% for G1 tumors and mitotic rate 2–20 and Ki‐67 3–20% for G2 tumors [7, 8]. G3 tumors appear as heterogeneous masses with areas of necrosis, lesser vascular density than lower‐grade tumors, and mitotic rate > 20, Ki‐67 > 20% [7, 8]. The upcoming classifications are expected to include additional mutation analyses, such as *DAXX*/*ATRX* mutations, to facilitate clinical management [7].

The majority (90%) of the PanNETs are regarded as sporadic and arise due to alterations that result in either loss of function in tumor suppressor genes or gain of function in oncogenes. The most frequently mutated genes within PanNETs are tumor suppressor genes *MEN1*, *DAXX*/*ATRX*, and genes related to mammalian target of rapamycin (mTOR) pathway (*PIK3CA*, *PTEN*, *DEPDC5*, *TSC2*). The commonly known tumor suppressor genes *TP53* and oncogenes *BRAF* and *KRAS* are usually intact in PanNETs, and the alterations are only found in poorly differentiated tumors (neuroendocrine carcinomas) [9, 10, 11, 12]. Recently, two genes have gained significant attention: *DAXX*/*ATRX*. Mutations in these genes have been identified in up to 40–43% of PanNET cases [7, 13]. Loss of nuclear DAXX/ATRX protein expression can promote alternative lengthening of telomeres (ALT), consequently leading to more aggressive tumor phenotypes. The 5‐year disease‐free survival and 10‐year disease‐specific survival rates for PanNETs with loss of DAXX/ATRX and ALT gained can be twice as low when compared to DAXX/ATRX positive PanNETs (50 and 40% vs. 89 and 96%) [13]. As a result, DAXX/ATRX and ALT analysis has recently gained attention as a valuable prognostic tool [13, 14].

One of the most significant components of any tumor is its microenvironment, also known as the tumor microenvironment (TME). It is a highly heterogeneous environment that consists of four major components: immune cells, stromal cells, blood vessels, and extracellular matrix [15]. Alongside the other components in TME, a vital role is played by the stroma, a compartment that mainly consists of fibroblasts, endothelial cells, and extracellular matrix [16]. The stromal cells can either hinder or promote tumor growth by upregulating angiogenesis, extracellular matrix remodeling, immune suppression, and altering the metabolism of the cells within TME [16]. Recent studies have shown that the upregulation of alpha‐smooth muscle actin (α‐SMA) in tumor stromal compartment correlates with worse outcomes and therapeutic resistance in various tumors [17, 18]. This could be attributed to the fact that α‐SMA is highly expressed by transformed fibroblasts known as cancer‐associated fibroblasts (CAFs) [19], which can harbor significant tumor‐promoting functions [20]. The molecular aspects of stroma have also been studied within NETs, where it has been shown that NET cells can recruit stromal fibroblasts by transforming them into α‐SMA expressing CAFs via the secretion of TGF‐β and platelet‐derived growth (PDGF) factors [21].

Due to the rarity of PanNETs, there are only a few studies that have characterized the overall transcriptomic landscape of PanNETs [22, 23]. Despite the evidence of existing crosstalk between stromal cells and NET cells, the underlying molecular mechanisms on the transcriptomic level remain largely understudied [21]. More so, the currently existing transcriptomic studies are limited by the bulk RNA sequencing approach, where the spatial and cellular composition information is lost [24]. Therefore, we employed GeoMx digital spatial profiling (DSP) in this study. A technology that allows us to extract the gene expression profiles from specific tissue compartments, which can be identified using morphology markers [25]. As a result, we characterized the gene expression profiles of 1800 cancer‐associated genes in PanNET cells, surrounding α‐SMA‐expressing (α‐SMA+) stromal cells, and islet/acinar compartments of the adjacent normal tissue. Furthermore, we also evaluated the differences in gene expression profiles between tumor cells that are located in regions with rich α‐SMA+ stromal cell presence and tumor cells that are in regions with poor α‐SMA+ stromal cell presence.

## 2 Materials and methods

### 2.1 Study cohort

Retrospective cases (2017–2021) of eight surgically resected non‐functioning primary sporadic PanNETs from two sites (Latvia and Spain) were investigated (Table 1). The cohort included five males and three females with a median age at the day of surgery – 58 years (range: 36–77 years). The study methodologies conformed to the standards set by the Declaration of Helsinki and accordingly were approved by the Central Medical Ethics Committee of Latvia (approval protocol No. 1.1–2/67) and Ramon y Cajal Ethical and Scientific Committees (protocol No. 196‐19). All patients were provided written informed consent before participation in this study and a physically signed version of the informed consent was obtained from each patient individually. Biological samples of PanNETs 6, 7, and 8 were collected by Ramón y Cajal University Hospital and further provided by the BioBank Hospital Ramón y Cajal‐IRYCIS (National Registry of Biobanks B.0000678), integrated into the Biobanks and Biomodels Platform of the ISCIII (PT20/00045). Biological samples of PanNETs 1, 2, 3, 4, and 5 were collected and obtained through Pauls Stradins Clinical University Hospital.

**Table 1:** Clinical characteristics of the patients recruited in the study. ANT, adjacent normal tissue; F, female; M, male; T, tumor tissue.

Sample ID	Sex	Age at surgery	Type of tissue	Grade	Ki‐67 index (%)	Stage	Mitotic index	Country of origin
PanNET1	M	65	T; ANT	G1	< 2%	II	1 mitoses/2 mm^2^	Latvia
PanNET2	F	58	T; ANT	G1	1%	I	< 2 mitoses/2 mm^2^	Latvia
PanNET3	F	52	T	G2	7–10%	I	3 mitoses/2 mm^2^	Latvia
PanNET4	M	44	T; ANT	G1	2%	II	1 mitoses/2 mm^2^	Latvia
PanNET5	M	70	T	G2	7–10%	II	5 mitoses/2 mm^2^	Latvia
PanNET6	F	36	T; ANT	G2	12%	III	9 mitoses/2 mm^2^	Spain
PanNET7	M	60	T; ANT	G2	5%	II	5 mitoses/2 mm^2^	Spain
PanNET8	M	77	T;ANT	G3	30%	IV	30 mitoses/2 mm^2^	Spain

All patients' tumor samples were synaptophysin positive, and none of the patients had received medical treatment before the surgery. Additional collected data included the patients' sex, grade and differentiation of the tumor, Ki‐67 index, stage of the disease, and patient age at surgery (Table 1). All cases were classified by tumor grade according to WHO 2019 criteria [26]. In total, three G1, four G2 patients, and one G3 patient were recruited, and FFPE samples of the tumor were obtained for DSP analysis. Six out of eight patients also had available FFPE samples of adjacent normal pancreatic tissues.

### 2.2 Immunohistochemistry

Additional markers such as ATRX/DAXX and PDX1/ARX were evaluated using immunohistochemistry after the DSP analysis to segregate tumors into molecular subgroups before statistical analyses. Immunohistochemistry (IHC) analysis was done by a pathologist at the Institute of Pathology laboratory at Pauls Stradins Clinical University Hospital. FFPE tissue samples were cut into 3‐μm‐thick sections. Deparaffinization was achieved using Dako PTLink station and EnVision FLEX Target Retrieval Solution HIGH pH (50×) solution (K8004, Agilent, Santa Clara, CA, USA) by incubating for 20 min at 96 °C followed by wash in EnVision FLEX WASH BUFFER (20×) solution (K800721‐2, Agilent). Staining was done on Autostainer Link 48 using EnVISION FLEX, High pH (Link) kit (K800021‐2, Agilent). The following antibodies were used: ATRX (D‐5) antibody (Mouse, sc‐55 584, SCBT, Dallas, TX, USA) diluted at 1 : 100; DAXX (H‐7) antibody (Mouse, sc‐8043, SCBT) diluted at 1 : 50; PDX1 (B‐11) antibody (sc‐390 792, SCBT), ARX antibody diluted at 1 : 100 (Rabbit, PA5‐109415, ThermoFisher, Waltham, MA, USA). Definitions of positive/negative expression were adopted from the Hackeng et al. study [27]. For ATRX/DAXX, the tumor was labeled positive if nuclear stain was present in > 5% of neoplastic cells. As for PDX1/ARX, the tumor was labeled positive if nuclear stain was present in > 10% of neoplastic cells. Pancreatic islet cells from adjacent normal tissue were used as a positive internal control for all four markers. Additionally, for ATRX/DAXX, lymphocytes were used as positive internal controls. The results of the IHC analysis are displayed in Table S1, and the representative samples showing either negative or positive expression are compiled in Fig. S1.

Overall, the ATRX/DAXX status was determined for five samples as these samples showed an apparent loss of nuclear ATRX expression with retained nuclear expression in positive internal controls. ATRX/DAXX status was undetermined for the remaining three samples. These samples were ATRX positive with undetermined DAXX expression due to the absence of positive internal controls. As for PDX1/ARX, all samples except PanNET4 retained positive ARX expression and lost PDX1 expression. PanNET4 sample was both PDX1/ARX negative. A noticeable heterogeneity was also observed amongst the ATRX and ARX positive samples, as not all neoplastic cells harbored positive nuclear expression. As such, we were unable to precisely assess whether the profiled regions were ATRX/ARX positive or negative. Based on these results, further statistical analyses were performed by grouping tumors only according to the tumor grade.

### 2.3 Slide preparation for DSP analysis

The slides were incubated for 3 h at 65 °C for paraffin removal and subsequently loaded onto a Leica BOND RX for tissue rehydration, heat‐induced epitope retrieval (ER2 for 20 min at 100 °C), and proteinase K treatment (0.1 μg·mL^−1^ for 15 min at 37 °C). The tissue sections were then hybridized with the Cancer Transcriptome Atlas (CTA) probes overnight at 37 °C. Following 2 × 5 min stringent washes (1 : 1 4× SSC buffer & formamide), the slides were blocked and then incubated with morphology marker antibodies to guide region of interest (ROI) selection: α‐SMA (488 channel, 53‐9760‐82, ThermoFisher), Synaptophysin (647 channel, ab196166, Abcam, Cambridge, UK), and Syto83 (532 channel, S11364, Invitrogen, Waltham, MA, USA)—a nuclear stain to assist with the segmentation. The stained sections were then analyzed on the GeoMx platform. A pathologist specializing in neuroendocrine tumors assisted with selecting the ROIs and confirmed the resulting AOIs before segmentation. For segmentation, UV light was directed by the GeoMx at each defined area of illumination (AOI), releasing the RNA ID and UMI‐containing oligonucleotide tags from the CTA probes for collection and preparation for sequencing. For samples PanNET1, 3, and 4, segmentation was based on synaptophysin fluorescent signal. For samples PanNET2, 5, 6, 7, and 8, which exhibited weak or non‐existent synaptophysin signal despite previously confirmed positivity by IHC, we performed a custom geometric segmentation guided by tissue morphology to target tumor cells specifically.

Illumina i5 and i7 dual indexing primers were added to the oligonucleotide tags during PCR to index each AOI uniquely. AMPure XP beads (Beckman Coulter, Brea, CA, USA) were used for PCR purification. Library concentration was measured using a Qubit fluorometer (Thermo Fisher Scientific), and quality was assessed using a Bioanalyzer (Agilent). Sequencing was performed on an Illumina NovaSeq 6000.

### 2.4 Processing and analysis of DSP data

The sequencing FASTQ files were processed using the GeoMx® NGS Pipeline to acquire GeoMX DSP Analysis Suite‐compatible DCC files. Following this, the DCC files were analyzed on the GeoMX DSP Analysis Suite, which included data quality control, filtering, normalization, and differential expression analysis. For the data quality control step, the reads from all 105 AOIs were inspected for raw reads aligned (cut‐off set at 80%), sequencing saturation (cut‐off set at 50%), negative probe count geomean (cut‐off set at five counts), and minimum nuclei count (cut‐off set at 200 nuclei). As a result, 78 AOIs were flagged for a negative probe count geomean below 5, six were flagged for an aligned read percentage below 70%, and 30 AOIs were flagged for a nuclei count below 200. Probe QC was also performed, and any probes that did not meet the following criteria: (geomean probe in all AOIs)/(geomean probes within target) ≤ 0.1, and failed Grubbs' outlier test in ≥ 20% of AOIs were removed from target count calculation. Following this, we also performed AOI and target filtering steps. For AOI filtering, any AOIs with less than 5% of targets above specific settings (higher of LOQ and user‐defined value of 2) expression threshold were filtered out. The filtering was also applied to targets, and any targets that were present in less than 3% of AOIs were removed from further analysis.

### 2.5 Statistical analyses

After the quality control and filtering steps, 466 genes and one AOI were excluded from further analysis, leaving a final raw count file containing information on 1482 genes and 104 AOIs, which was used for the downstream analysis (Table S2). The raw count data were then normalized using the Q3 normalization method in GeoMX DSP Analysis Suite (Table S3). t‐SNE‐based dimension reduction analysis was performed on Q3 normalized data using the GeoMx DSP analysis suite plugin – DimensionReduction (v1.1). To determine cell type abundance in analyzed AOIs and to estimate genes of interest correlation with cell type abundances, a spatial deconvolution analysis was performed in r (v4.2.2) using a Bioconductor package spatialdecon (v1.8.0) with the inbuilt SafeTME cell profile matrix [28]. In deconvolution analysis, tumor AOIs from PanNET1, PanNET4, and PanNET8 samples were used to update the SafeTME matrix with tumor cell gene expression profiles derived from this study.

Linear mixed model analysis was performed in GeoMx DSP Analysis Suite to analyze differential gene expression between different AOI groups. For all analyses, the sample preparation batch was used as a covariate (ScanID column in Table S4). Volcano plots depicting the results were made using the GeoMx Analysis Suite plugin – Volcano Plot (v1.2).

We also evaluated whether the two different segmentation strategies introduced a possible batch effect by performing clustering and PCA analysis. The analysis was done in r (v4.4.0). Before the PCA and clustering analysis, the data containing Q3 normalized counts from 45 tumor and 18 islet AOIs were imported in r, transformed using the Log_2_(count +1) method, and corrected for a ScanID batch effect. For batch effect correction, limma package (v3.60.2) was used. Following this, the PCA analysis was performed, and hierarchical clustering analysis was performed using the pheatmap package (v1.0.12). The results indicate that samples cluster together according to tumor grade (Fig. S2). Moreover, it can be observed that AOIs from the same tumor sample are similar to one another, and there are observable differences between tumor samples regardless of tumor grade. No substantial batch effect is observed due to the segmentation strategy. As no substantial batch effect was detected, the models were not further adjusted.

### 2.6 Pathway enrichment and protein–protein interactions analyses

DEGs with Log_2_FC > 0.5 and FDR < 0.05 values were subjected to pathway enrichment analysis and protein–protein interaction (PPI) analysis using the STRING database (v11.5) [29] and stringApp plugin (v2.0.1) in Cytoscape (v3.9.1). A whole genome background was used for STRING analysis, and the minimum required interaction score was set at 0.4. Dot plots depicting the enriched pathways were made using r (v4.2.2) package ggplot2 (v3.4.0), and PPI networks were constructed in Cytoscape using additional plugins: networkanalyzer (v4.4.8), legendcreator (1.1.6) and enhanced graphics (1.5.5).

## 3 Results

### 3.1 Overview of AOIs analyzed by the digital spatial profiler

**Fig. 1:**
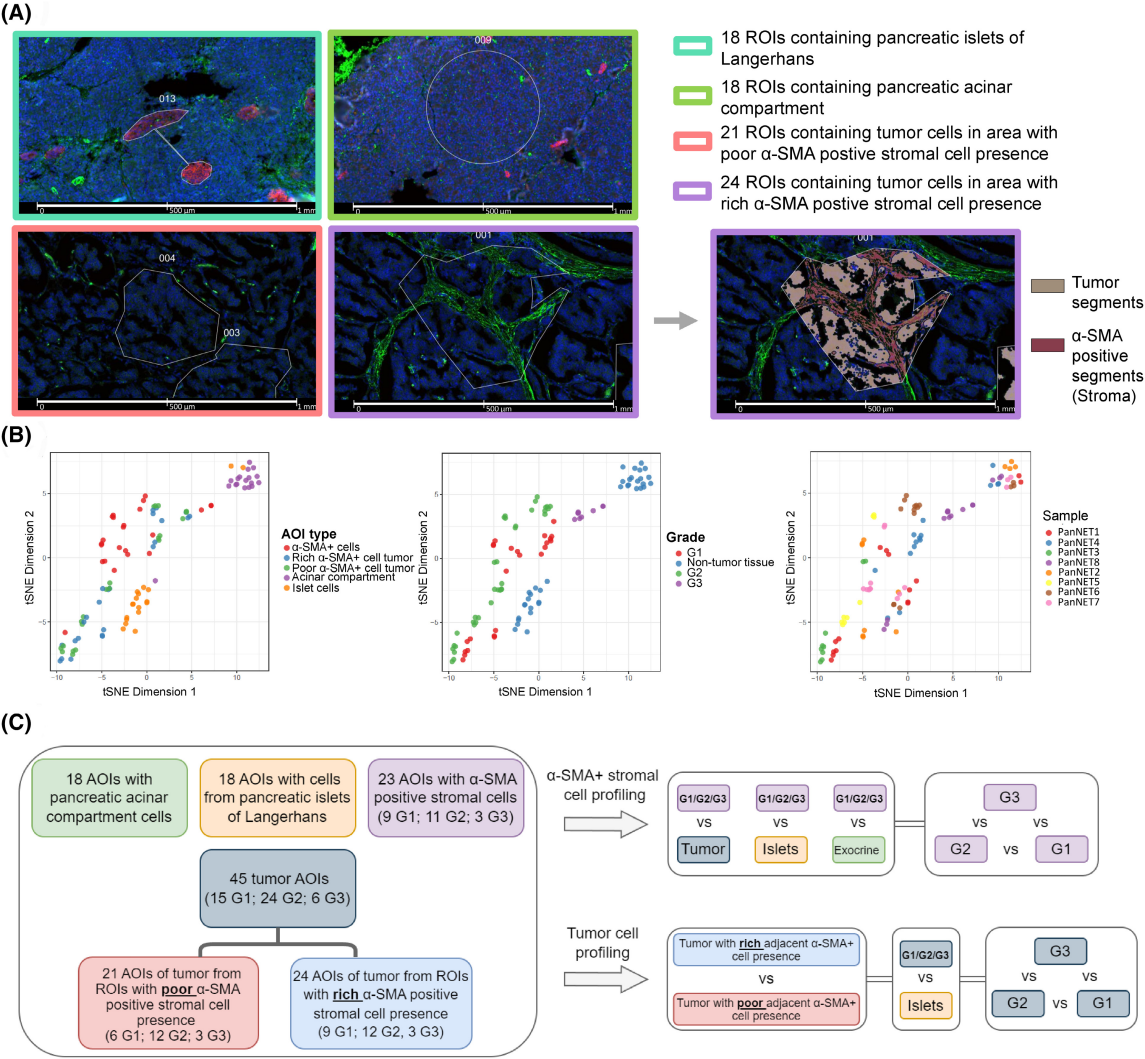
Study design for digital spatial profiling of three grade 1 (G1), four grade 2 (G2), and one grade 3 (G3) pancreatic neuroendocrine tumor tissue samples. (A) Represents the strategy used to select regions of interest (ROI) and areas of illumination (AOI). Scale bar in all four images—1000 μm. ROIs containing islets of Langerhans (18×) and acinar compartment (18×) within adjacent normal tissues were selected based on the presence of synaptophysin and morphology; no further segmentation was performed, and AOI count was the same as the ROI count (36×). Within the tumor, ROI selection was based on the presence of alpha‐smooth muscle actin‐expressing (α‐SMA+) stromal cells. As a result, 21 ROIs with tumors containing a poor or absent adjacent α‐SMA+ cell presence were selected, yielding 21 tumor AOIs. Following this, another 24 ROIs were selected, where a rich α‐SMA+ stromal cell presence adjacent to the tumor was observed. Here, segmentation was performed based on the α‐SMA and synaptophysin signals or α‐SMA signal, assisted by custom geometric‐based segmentation, in case tumor cells did not exhibit synaptophysin signal. This yielded 24 α‐SMA+ stroma adjacent tumor AOIs and 24 AOIs containing α‐SMA+ stromal cells. (B) T‐distributed neighbor embedding (t‐SNE) dimensionality reduction using Q3 normalized count data from all 104 sequenced AOIs. t‐SNE analysis was based on the top 500 genes with the highest variance. (C) Flowchart depicting the study design of the differential expression analyses with all 104 AOIs. The tumor and α‐SMA+ stromal AOIs were grouped according to tumor grade and then compared using linear mixed model analysis.

Based on immunofluorescence and morphology, we selected 45 regions of interest (ROIs) within eight tumor tissue samples (detailed information on each patient and tumor sample can be found in Table 1). ROI selection was based on the abundance of α‐SMA+ stromal cells in close proximity to the tumor cells. As a result, 24 ROIs contained tumor cells with rich adjacent α‐SMA+ stromal cell presence, and 21 ROIs contained tumor cells with poor adjacent α‐SMA+ stromal cell presence. The tumor ROIs containing both α‐SMA+ cells and tumor cells were segmented into two AOIs. This yielded 24 AOIs containing tumor cells and 23 AOIs containing α‐SMA+ stromal cells (one AOI was dropped due to a low number of targets above the defined expression threshold) (Fig. 1A). This amounted to a total of 68 AOIs from 45 ROIs within tumor tissue samples. Metadata and images of all 45 and 68 profiled ROIs/AOIs in tumor samples can be found in Tables S4 and S5, respectively. Using DSP, we also investigated normal pancreatic tissue and selected 18 ROIs containing Islets of Langerhans and 18 ROIs containing acinar compartment, which amounted to a total of 36 AOIs from 36 ROIs. As a result, the gene expression was assessed in 104 AOIs. By applying the T‐distributed neighbor embedding (t‐SNE) dimensionality reduction, we can observe segregation by AOI type as a distinction between tumor, α‐SMA+ stroma, and non‐tumor cell AOIs can be observed (Fig. 1B). Following this, the gene expression data of all 68 tumor AOIs were subjected to cell type deconvolution analysis to estimate the relative abundance of cell types within each AOI. The proportion of fitted cells (Fig. S3) shows that α‐SMA+ stroma AOIs have a higher proportion of stromal cells than tumor AOIs. As expected, within the tumor‐specific AOIs, the most abundant cell type was PanNET cells. We also observed the second most abundant cell type within tumor tissue was T cells. A complete list of cell type counts and proportions in tumor tissues can be found in Table S6. Design of further differential expression analyses is depicted in Fig. 1C.

### 3.2 Identification of genes associated with SMA+ stroma compartments

**Fig. 2:**
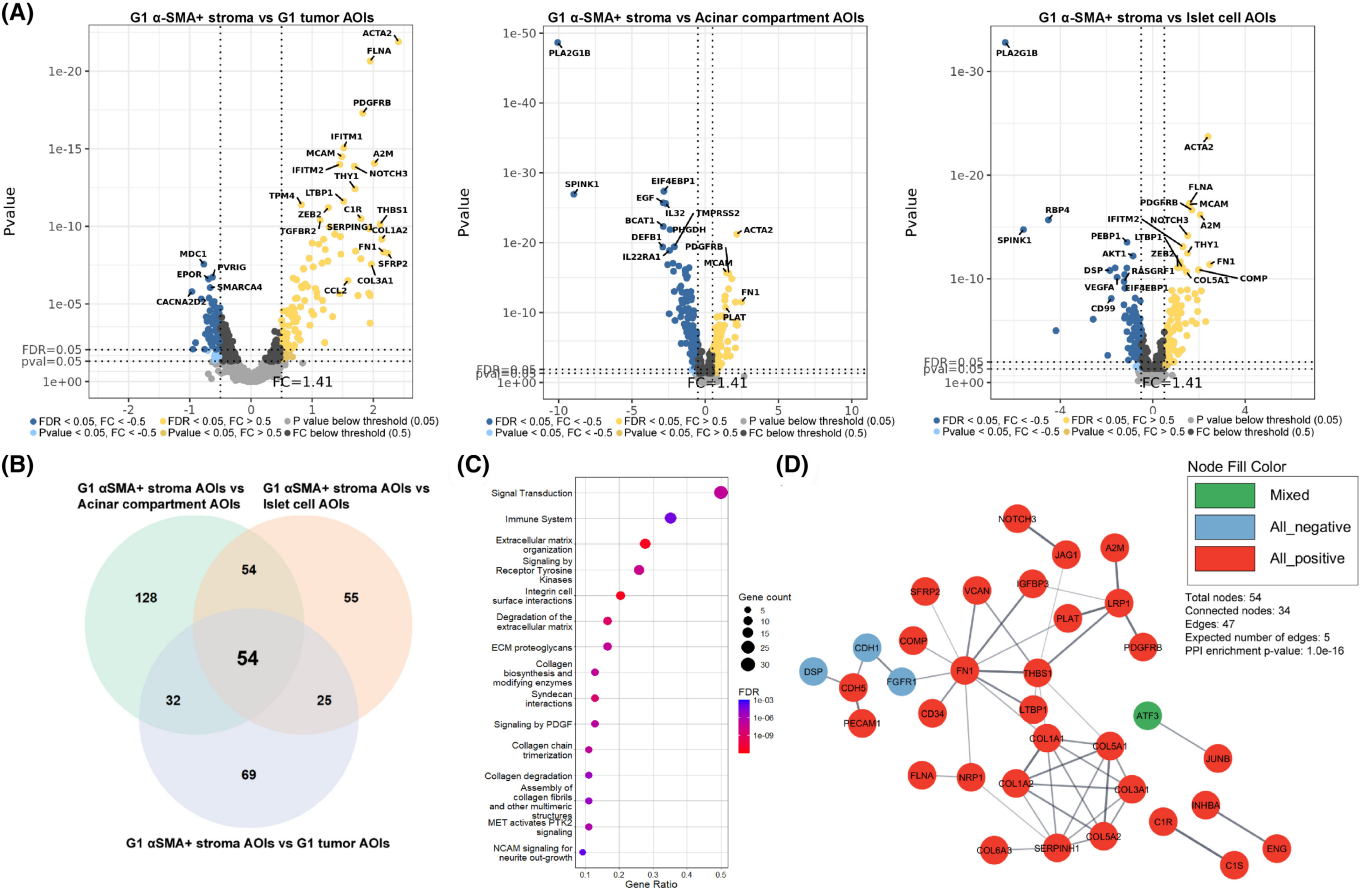
Analysis of alpha‐smooth muscle actin‐expressing (α‐SMA+) stromal cell areas of illumination from grade 1 tumor tissues. (A) volcano plots depicting the Log_2_ fold changes (*x*‐axis) and *P*‐values (*y*‐axis) from differential expression analyses where a set of nine α‐SMA+ stromal cell areas of illumination (AOIs) from grade 1 (G1) tumors were compared against sets of 15 G1 tumor, 18 acinar compartment, and 18 islet cell AOIs. (B) Venn diagram depicting a set of 54 overlapping differentially expressed genes between all three comparisons, which were used as an input for STRING enrichment analysis. (C) Dot plot depicting the top 15 (according to false discovery rate – FDR value) overrepresented Reactome pathways. Terms are sorted by the count of genes related to the specific term. (D) STRING network of physical protein associations showing physical interactions between proteins of input genes.

To identify genes specific to α‐SMA+ stroma within PanNET tissues, we carried out differential expression analysis where α‐SMA+ stroma AOIs of each tumor grade were compared to tumor AOIs and acinar compartment/islet cell AOIs from normal tissue. After the differential expression analyses, we identified the sets of overlapping genes between these comparisons to further elucidate the genes that are associated explicitly with α‐SMA+ stromal cells (Table S7). As a result, in the comparisons of α‐SMA+ AOIs from G1 tumor (Fig. 2A), we identified a set of 54 overlapping genes (Fig. 2B), of which 42 had a positive, seven had a negative, and five had a mixed fold change direction in all three comparisons. Further subjecting a set of 54 overlapping genes to STRING functional enrichment analysis showed 32 overrepresented Reactome pathways (Table S8). The top 15 pathways are listed in Fig. 2C. To further expand on the interactions between overlapping genes, a STRING network of physical protein associations was constructed, which revealed 47 interactions between 34 proteins with protein–protein interaction (PPI) enrichment *P*‐value of 1.0 × 10^−16^ (Fig. 2D).

**Fig. 3:**
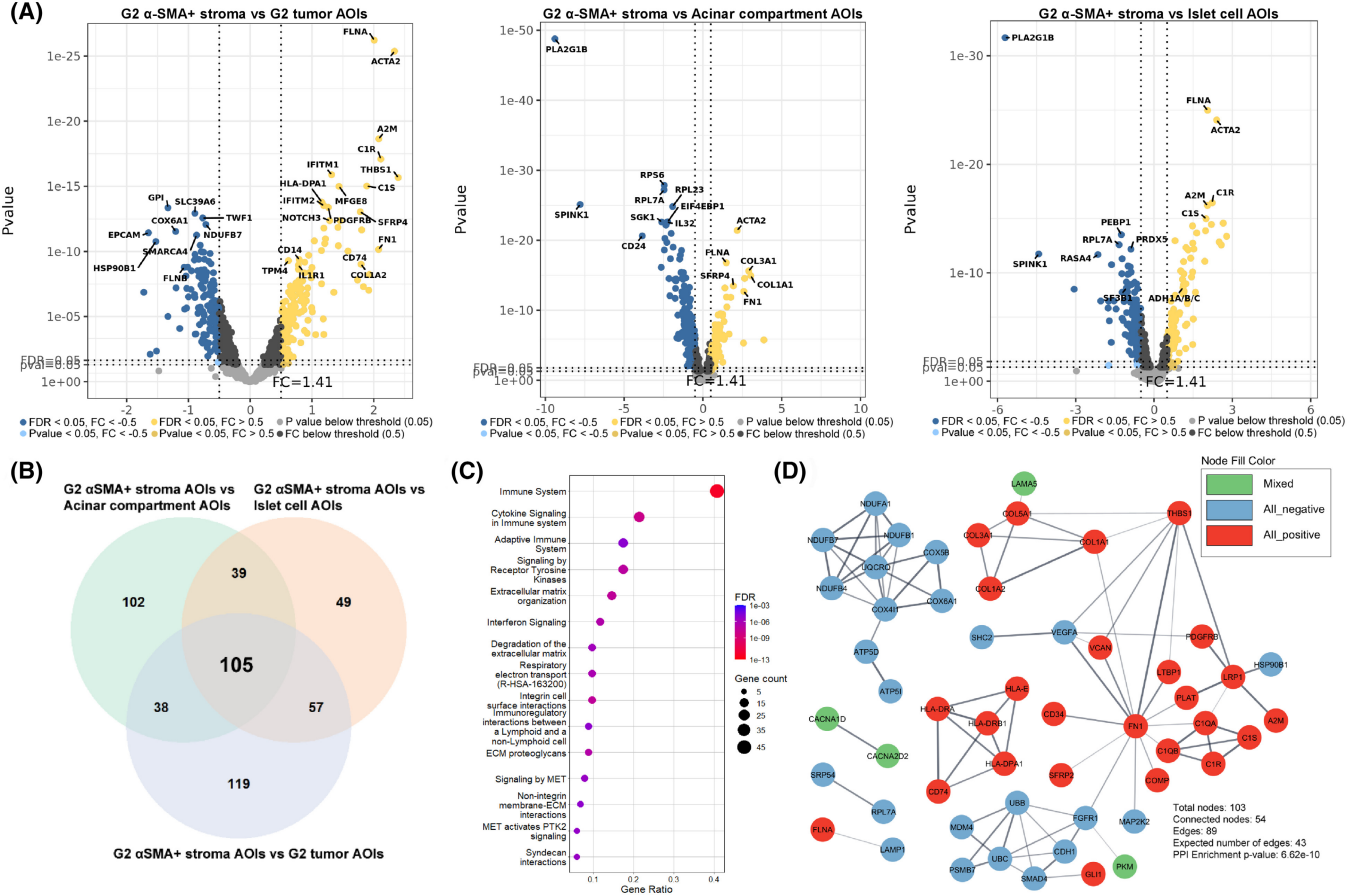
Analysis of alpha‐smooth muscle actin‐expressing (α‐SMA+) stromal cell areas of illumination from grade 2 tumor tissues. (A) volcano plots depicting the Log_2_ fold changes (*x*‐axis) and *P*‐values (*y*‐axis) from differential expression analyses where a set of 11 α‐SMA+ stromal cell areas of illumination (AOIs) from grade 2 (G2) tumors were compared against sets of 24 G2 tumor, 18 acinar compartment, and 18 islet cell AOIs. (B) Venn diagram depicting a set of 105 overlapping differentially expressed genes between all three comparisons, which were used as an input for STRING enrichment analysis (2 genes were excluded due to missing records in the STRING database). (C) Dot plot depicting the top 15 (according to false discovery rate – FDR value) overrepresented Reactome pathways. Terms are sorted by the count of genes related to the specific term. (D) STRING network of physical protein associations showing physical interactions between proteins of input genes.

**Fig. 4:**
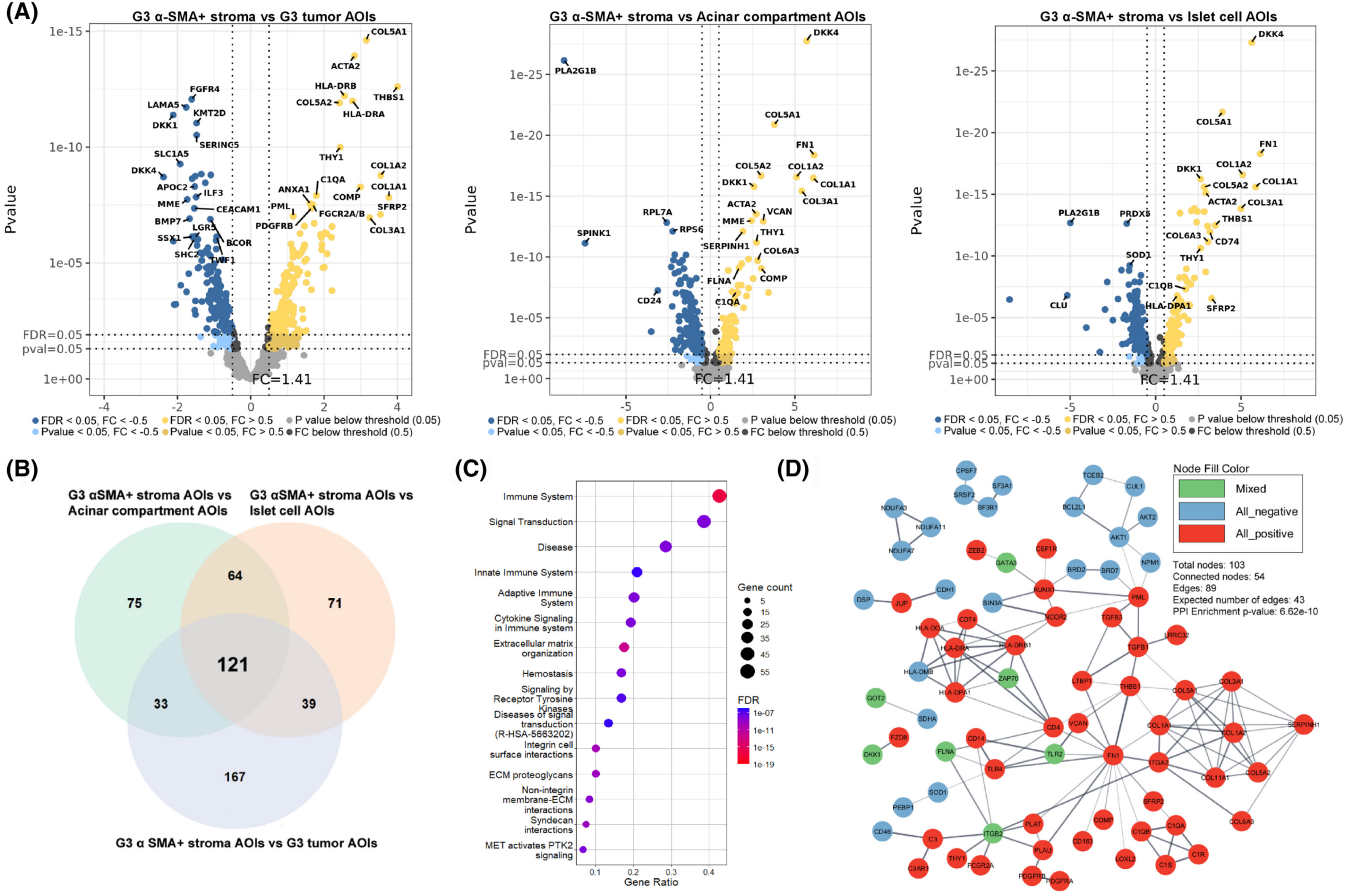
Analysis of alpha‐smooth muscle actin‐expressing (α‐SMA+) stromal cell areas of illumination from grade 3 tumor tissues. (A) volcano plots depicting the Log_2_ fold changes (*x*‐axis) and *P*‐values (*y*‐axis) from differential expression analyses where a set of three α‐SMA+ stromal cell areas of illumination (AOIs) from grade 3 tumor were compared against sets of six G3 tumor, 18 acinar compartments, and 18 islet cell AOIs. (B) Venn diagram depicting a set of 121 overlapping differentially expressed genes between all three comparisons, used as an input for pathway enrichment analysis (2 genes were excluded due to missing records in the STRING database). (C) Dot plot depicting the top 15 (according to false discovery rate – FDR value) overrepresented Reactome pathways. Terms are sorted by the count of genes related to the specific term. (D) STRING network of physical protein associations showing physical interactions between proteins of input genes.

In the G2 scenario (Fig. 3A), 105 overlapping genes were found (Fig. 3B), which was substantially higher than in the G1 scenario. Similarly to the G1 scenario, we also observed a uniform fold change direction of the overlapping genes as 53 genes showed positive, 41 negative, and 11 mixed fold change directions in all three comparisons. Here, the STRING enrichment analysis revealed 190 overrepresented Reactome pathways (Table S8). The top 15 pathways are listed in Fig. 3C. In the network of physical protein associations, 89 interactions were found between 54 proteins (Fig. 3D) with a *P*‐value of 6.622 × 10^−10^, indicating that proteins of overlapping genes function as a biological group. Lastly, within the G3 comparison scenario (Fig. 4A), the number of overlapping genes (Fig. 4B) was the highest (121 genes). There was also a uniform fold change direction across all three comparisons as 71 genes showed positive, 35 negative, and 15 mixed fold change directions in all three comparisons. Amongst the list of 121 overlapping genes, 90 overrepresented Reactome pathways were discovered (Table S8), and the top 15 pathways are listed in Fig. 4C. Within the network of physical protein associations (Fig. 4D), the interactions were more pronounced compared to the G2 overlapping gene network as 137 interactions were observed between 77 proteins with a *P*‐value of 1 × 10^−16^.

In all three scenarios, the top 15 pathways associated with the overlapping DEGs were mainly related to the events of the immune system, extracellular matrix organization, and MET signaling (Figs 2C, 3C and 4C). Upon further inspection of STRING protein networks, we observed that in G2 and G3 tumors, the immune system pathways were especially pronounced as a cluster of tightly interacting genes related to the immune system were found: *CD74*, *HLA‐E*, *HLA‐DRA*, *HLA‐DPA1*, *HLA‐DRB1* in G2 (Fig. 3D) and *CD74*, *HLA‐DRA*, *HLA‐DPA1*, *HLA‐DRB1*, *HLA‐DOA*, *HLA‐DMB*, *ZAP70* in G3 (Fig. 4D). Following this, we also identified clusters containing a multitude of tightly interacting collagen genes which were upregulated in α‐SMA+ stroma across all three grades: *COL1A1*, *COL5A1*, *COL1A2*, *COL3A1*, *COL5A2* in G1 (Fig. 2D), *COL1A1*, *COL5A1*, *COL1A2*, *COL3A1* in G2 (Fig. 3D), *COL1A1*, *COL5A1*, *COL1A2*, *COL3A1*, *COL5A2*, *COL6A3*, *COL11A* in G3 (Fig. 4D). The pathway enrichment analysis shows that these genes are exactly involved in pathways regarding extracellular matrix organization and MET signaling events. Combined with the aforementioned immune‐related genes, collagen genes were also involved in immune system pathways. It can also be observed that all the collagen proteins are strongly linked to the *SERPINH1* gene (Figs 2D and 4D), which, according to the STRING database, is a serine protease inhibitor and plays a role in collagen processing by acting as a chaperone.

**Fig. 5:**
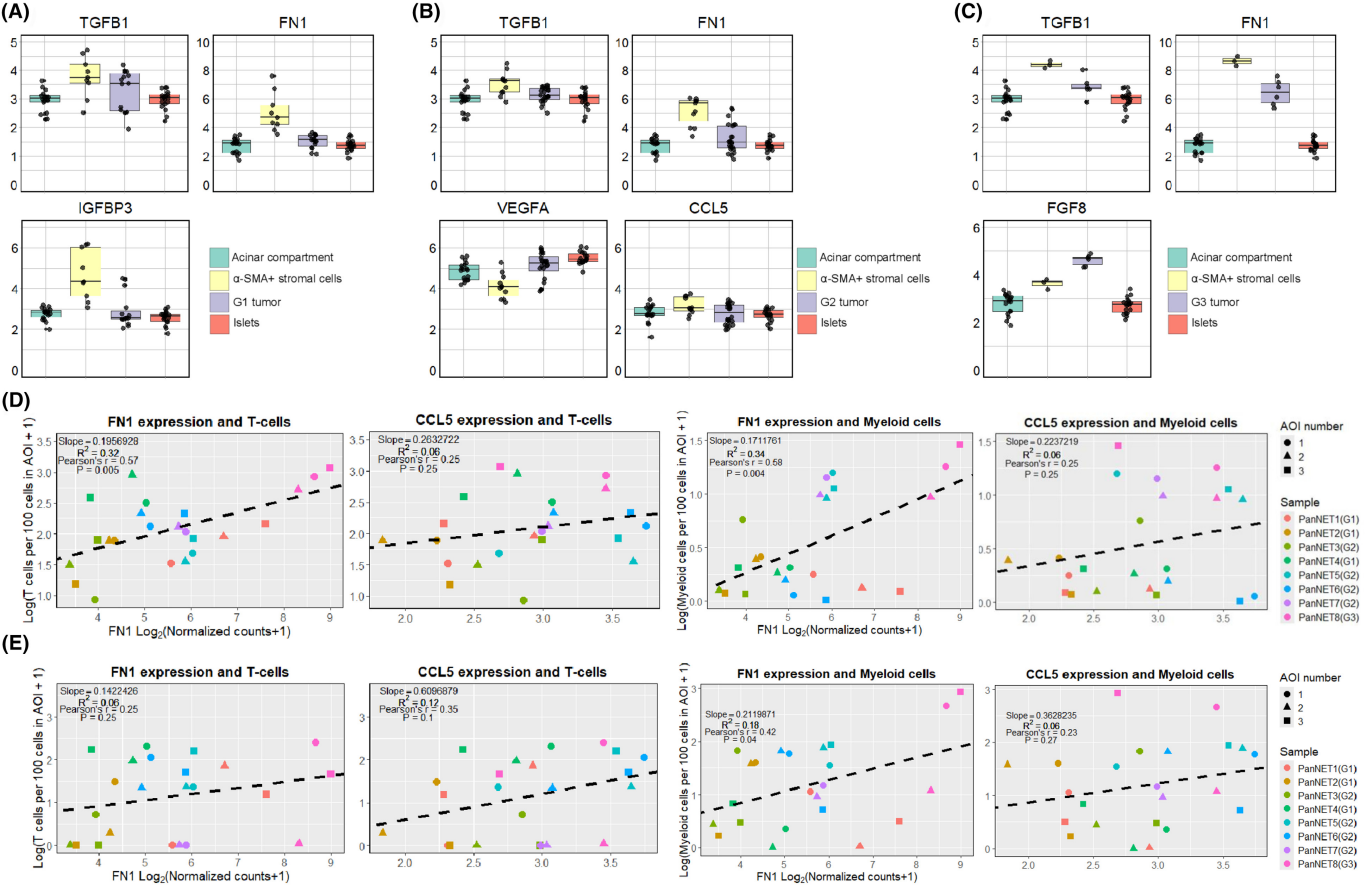
The expression of genes related to the secretome of cancer‐associated fibroblasts in alpha‐smooth muscle actin‐expressing areas of illumination. (A–C) Box plots that represent the genes that are related to cancer‐associated fibroblast (CAF) secretome and are differentially expressed in all three comparisons: α‐SMA+ stroma versus (1) tumor, (2) acinar compartment, and (3) islet cell areas of illumination (AOIs). The *Y*‐axis of boxplots represents Log_2_(Normalized counts +1). (A) Grade 1 tumors, (B) grade 2 tumors, (C) grade 3 tumors. (D, E) Scatter plots with linear regression trendlines representing a correlation between *FN1*/*CCL5* gene expression and deconvoluted T‐cell/myeloid cell counts. The analysis was done using the Q3 normalized count data and the resulting cell counts per 100 cells in AOI data from SpatialDecon (v1.8.0) analysis. The *X*‐axis represents Log_2_(Normalized counts +1) of the target gene, and the *Y*‐axis represents Log(Cell counts per 100 cells in AOI +1) of the target cell type. (D) Tumor AOIs from ROIs with rich adjacent α‐SMA+ stromal cell presence, (E) α‐SMA+ stromal cell AOIs.

Lastly, we also looked at the expression of various ligands secreted by stromal cells, particularly the CAF component of stroma, which has been listed in multiple secretome studies and reviews [30, 31, 32, 33, 34, 35, 36]. Here, we also focused on reporting only those factors that were differentially expressed (FDR < 0.05) in all three comparisons. Across all three tumor grades, we observed upregulation of two factors: *TGFB1* (TGF‐β) and *FN1* (fibronectin) in α‐SMA+ stroma AOIs when compared to all other tissue type AOIs (Fig. 5A–C). Additionally, in the G1 and G3 groups, one additional factor was upregulated: *IGFBP3* (IGF‐Binding Protein 3) in the G1 group and *FGF8* (fibroblast growth factor 8) in the G3 group. In the G2 group, two additional factors were differentially regulated where: *CCL5* (RANTES) was upregulated, and *VEGFA* (vascular endothelial growth factor) was downregulated in α‐SMA+ stromal cells.

Furthermore, some studies have shown that *FN1* and *CCL5* [37, 38, 39] gene expression positively correlates with immune cell infiltration within the tumor. Therefore, we performed a linear regression and Pearson correlation analysis to investigate this relationship in our samples with *FN1* expression data and cell count data acquired from deconvolution analysis. As a result, we observed that *FN1* expression in α‐SMA+ stroma had a statistically significant positive correlation with both myeloid cell and T cell levels in tumor AOIs from ROIs with rich α‐SMA+ stromal cell presence (Fig. 5D). We also evaluated the correlation in α‐SMA+ stromal cell AOIs, but a statistically significant correlation was observed only between *FN1* and myeloid cell levels (Fig. 5E). As for the *CCL5* gene, although positive correlations were observed in all scenarios, none of them were statistically significant.

### 3.3 Comparison of α‐SMA+ stroma compartments across tumor grades

**Fig. 6:**
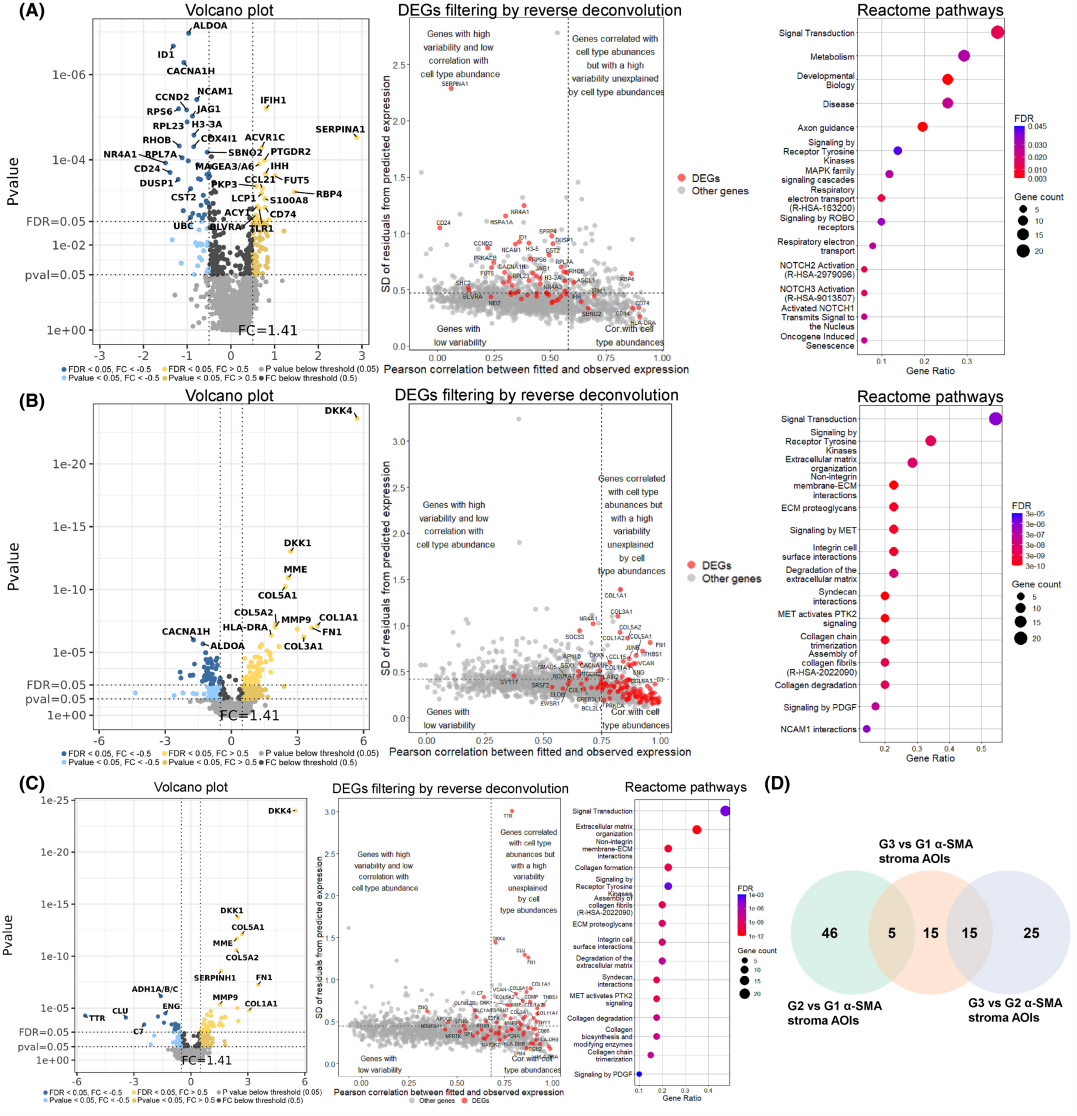
Comparison of alpha‐smooth muscle actin‐expressing (α‐SMA+) areas of illumination (AOIs) between different tumor grades. (A–C) Figures contain (1) volcano plots depicting the Log_2_ fold changes (*x*‐axis) and *P*‐values (*y*‐axis) of the assessed genes. (2) Scatterplots (DEGs filtering by reverse deconvolution) that display the results from Reverse Deconvolution where the *x*‐axis represents Pearson correlation between observed and fitted expression (based on cell abundance) and the *x*‐axis represents the standard deviation of Log_2_‐transformed residuals. Vertical and horizontal lines show third quartile (Q3) values used as cut‐offs. Any genes with correlation scores above Q3 and residual standard deviation values below Q3 were excluded from further STRING enrichment analysis. (3) Dot plots (Reactome pathways) of the top 15 Reactome pathways according to false discovery rate (FDR) value. The terms are sorted by gene count related to the term. (A) Analysis of 11 grade 2 (G2) vs. nine grade 1 (G1) AOIs; (B) Analysis of three grade 3 (G3) vs. nine G1 AOIs; (C) Analysis of three G3 vs. 11 G2 AOIs. (D) Venn diagram depicting the overlapping DEGs between the three comparisons.

Our next goal was to understand the differences in gene expression profiles of AOIs targeting α‐SMA+ stromal cells between different tumor grades. As a result, we carried out differential expression analysis where α‐SMA+ AOIs were compared against each other depending on the tumor grade (G1, G2, G3) (Table S9). Given the observable differences in the relative abundances of stromal and other cell types within each individual a‐SMA+ AOI, we conducted reverse deconvolution analysis to determine whether the variability in specific DEGs is driven by differences in cell type abundances or due to differential regulation within the same cell type. Following that, any genes with Pearson correlation values above the Q3 value and residual standard deviation values below the Q3 value were excluded from the downstream analysis. When G2 tumor α‐SMA+ AOIs were compared to G1, a total of 57 DEGs (22 upregulated, 35 downregulated) were identified at FDR < 0.05 and Log_2_FC > 0.5 cut‐offs (Fig. 6A). The reverse deconvolution analysis showed that the majority of the DEGs (51 DEGs: 18 upregulated and 33 downregulated) do not show strong associations with cell type abundances and are rather differentially regulated within the same cell types. By subjecting these 51 DEGs to STRING enrichment analysis, we identified 14 enriched Reatcome pathways (Fig. 6A, Table S10). Amongst these results, we found three notable terms related to NOTCH signaling, which included genes (*JAG1*, *APH1B*, and *UBC*). The STRING network of physical protein associations showed a relatively low number of physical associations between the proteins of these 51 DEGs, as the network consisted of 14 interactions between 19 nodes with a PPI enrichment *P*‐value of 0.368 (Fig. S4A). When we compared the G3 tumor to both G1 and G2 tumors, a total of 109 (66 upregulated, 43 downregulated) and 63 (45 upregulated, 18 downregulated) DEGs were found. However, as the cell type proportions of the G3 α‐SMA+ AOIs were substantially different from G1 and G2 tumors α‐SMA+ AOIs, the majority of DEGs showed strong associations with differences cell type abundances between analyzed AOIs (Fig. 6B,C). As a result, in the G3 vs. G1 comparison, 35 out of 109 DEGs (16 upregulated, 19 downregulated) and in the G3 vs G2 comparison, 40 out of 63 DEGs (27 upregulated, 13 downregulated) were subjected to further downstream analysis. As a result, an overrepresentation of 39 Reactome pathways was found in the list of 35 filtered DEGs from the G3 vs G1 comparison. As for 40 filtered DEGs from the G3 vs G2 comparison, we identified 31 overrepresented Reactome pathways (Table S10). The top 15 enriched pathways were associated with extracellular matrix organization in both comparisons. Moreover, in both scenarios, all of the top 15 overrepresented pathways included multiple physically interacting (on protein level) collagen genes, which were all upregulated in G3 tumor α‐SMA+ segments (Fig. S4B,C). It is necessary to mention that in both comparisons with the G3 tumor, the collagen genes also show very strong correlations with cell type abundances (*r* > 0.8). However, as demonstrated by their standard deviance of residuals these genes still have substantial variability unexplained by cell type abundances. Lastly, the Venn diagram of DEGs (Fig. 6D) shows that the α‐SMA+ cells of G3 tumor have a gene expression profile that is distinct from both G2 and G1 tumors; however, again, this could be mainly due to cell mixing, as depicted by reverse deconvolution analyses.

### 3.4 Comparison of tumor regions with abundant α‐SMA+ stromal cell presence versus regions with poor α‐SMA+ stromal cell presence

**Fig. 7:**
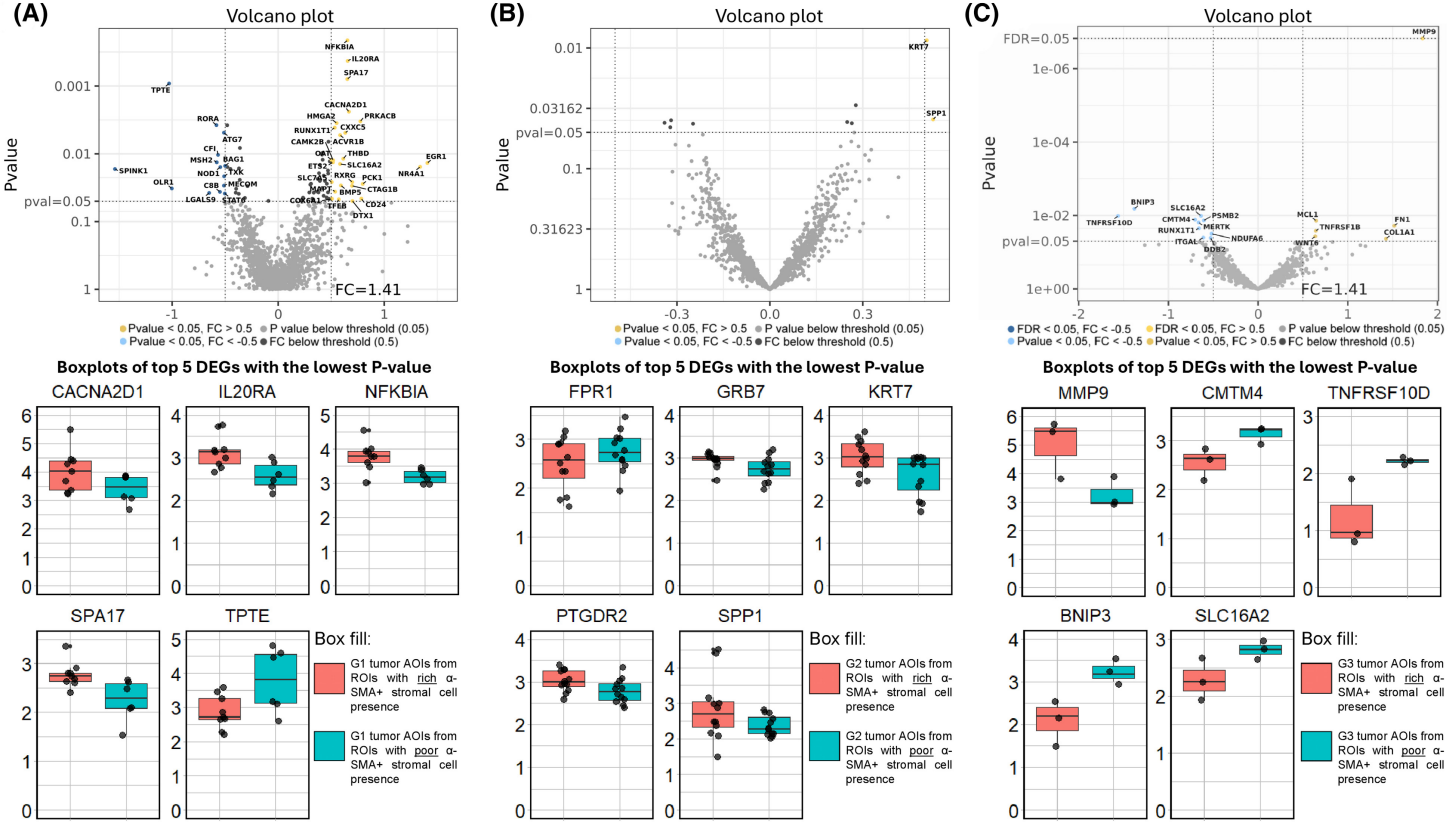
Results of differential expression analysis between tumor areas of illumination (AOIs) from regions of interest (ROIs) with rich alpha‐smooth muscle actin‐expressing (α‐SMA+) stromal cell presence vs. tumor AOIs from ROIs with low/absent α‐SMA+ stromal cell presence. The analysis was done across all three tumor grades. The results contain (1) volcano plots depicting the Log_2_ fold changes (*x*‐axis) and *P*‐values (*y*‐axis) of the assessed genes. (2) The boxplots depicting the Log_2_ (Normalized counts +1) transformed normalized count data of five differentially expressed genes with the lowest *P*‐value. (A) Grade 1 (G1) tumors comparison, (B) Grade 2 (G2) tumors comparison, (C) Grade 3 (G3) tumor comparison.

Another goal of this study was to assess whether there are differences in gene expression profiles between tumor cells in regions where the tumor has a rich α‐SMA+ stromal cell presence and regions with poor α‐SMA+ stromal cell presence. As a result, tumor AOIs from ROIs encompassing both tumor and α‐SMA+ stromal cells were compared against tumor AOIs from ROIs that were distant from any α‐SMA+ stroma cells. Images of investigated tumor ROIs and the resulting AOIs after segmentation can be found in Table S5. Across all three grades, the differences in gene expression were marginal (Fig. 7). At the FDR cut‐off at 0.05 and Log_2_FC cut‐off at 0.5, only one DEG was found (*MMP9*) in a G3 tumor, which was upregulated in tumor AOIs from ROIs with rich α‐SMA+ stromal cell presence. By taking into account only the unadjusted *P*‐values and without Log_2_FC cut‐off in G1 tumors, differences were observed for 81 genes (55 upregulated, 26 downregulated). In G2 tumors, the differences were far less pronounced, as a difference in expression was observed only for nine genes. Lastly, in the G3 tumor, aside from *MMP9*, 15 additional genes (five upregulated, 10 downregulated) had a *P*‐value of < 0.05 (Table S11).

### 3.5 Identification of altered genes in PanNET cells in comparison to pancreatic islet cells

**Fig. 8:**
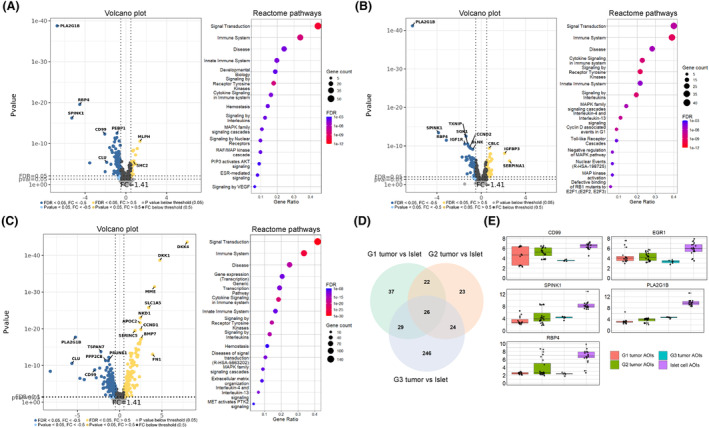
Comparison of tumor cell areas of illumination (AOIs) against pancreatic islet cell AOIs across all three tumor grades. (A–C) Figures contain (1) volcano plots depicting the Log_2_ fold changes (*x*‐axis) and *P*‐values (*y*‐axis) of the assessed genes, (2) dot plots of the top 15 Reactome pathways according to FDR value, which are sorted by gene count related to the term. (A) 15 grade 1 (G1) tumor AOIs vs. 18 islets AOIs; (B) 24 grade 2 (G2) tumor AOIs vs. 18 islet AOIs; (C) 6 grade 3 (G3) tumor AOIs vs. 18 islet AOIs. (D) Venn diagram depicting the overlapping DEGs between the three comparisons. (E) Boxplots of five consistently downregulated genes across all three tumor grades. *X*‐axis represents Log_2_ (Normalized counts +1).

To identify genes that are specifically associated with PanNET cells, we compared the AOIs containing PanNET cells against AOIs containing pancreatic islets of Langerhans (Table S12). Since PanNETs arise from the islet cells, this allows us to more precisely assess the changes that are related to tumorigenesis rather than to the difference in cell types. In the list of DEGs with FDR < 0.05 and Log_2_FC > 0.5 (volcano plots in Fig. 8A–C), the first notable finding here is that amongst all three grades, we can observe 26 overlapping DEGs (Fig. 8D) of which five genes (*RBP4*, *SPINK1*, *PLA2G1B*, *EGR1*, *and CD99*) showed a consistent downregulation (Fig. 8E). Additionally these were also amongst the top 10 downregulated genes in all three comparisons. By looking at each comparison individually at FDR < 0.05 and Log_2_FC > 0.5 thresholds in G1 tumor AOIs comparison against islet cell AOIs, we identified 114 differentially expressed genes (89 downregulated, 25 upregulated), which showed statistically significant associations with 51 Reactome pathways in STRING enrichment analysis (Table S13). Amongst the top 15 pathways (Fig. 8A), we found RAF/MAP kinase cascade, MAPK signaling cascades, and PIP3 activates AKT signaling, which are all linked to cell proliferation and survival. Another unique pathway was signaling by VEGF, which is involved in angiogenesis and has been extensively studied in neuroendocrine tumors [40]. The majority of DEGs related to this pathway (*ITGAV*, *KDR*, *VEGFB*, *AKT1*, *VEGFA*) were downregulated in tumor cells except for *HSBP1* and *SHC2*. Further analyzing physical interactions between DEGs, a STRING network of physical protein associations showed 75 interactions between 64 proteins with an enrichment *P*‐value of 4.88 × 10^−9^ (Fig. S5A).

In G2 tumors, the number of DEGs was slightly less than in G1 tumors (95 DEGs, 26 upregulated, and 69 downregulated). In enrichment analysis, these DEGs showed statistically significant associations with 168 Reactome pathways (Table S13). Interestingly, the two downregulated genes that were related to the ubiquitin system (*UBC* and *RPS27A*) were present in more than half of 168 pathways, 116 and 131, respectively. Amongst the top 15 pathways (Fig. 8B), we found multiple pathways related to cell proliferation, differentiation, and survival (MAPK pathways, cyclin D‐associated events in G1, defective binding of RB1 mutants to EF1). Amongst the 168 pathways, we did not detect pathways associated with VEGF signaling, which was prevalent in G1 tumors, as none of the DEGs related to VEGF signaling in G1 tumors were differentially expressed in G2 tumors. In a network of physical protein associations, 53 interactions between 37 proteins were found with a PPI enrichment *P*‐value of 0.01 (Fig. S5B).

In the G3 tumor, the number of DEGs was the highest as 325 genes (152 upregulated, 173 downregulated) were dysregulated. Here, enrichment analysis showed 320 statistically significant Reactome pathways (Table S13). Amongst these were three pathways related to VEGF signaling, which constituted of four upregulated (*MAPK14*, *HSPB1*, *SHC2*, *FLT1*) and six downregulated (*KDR*, *PRKACA*, *PAK3*, *PRKACB*, *RHOA*, *VEGFA*) genes. Within the top 15 pathways (Fig. 8C), the MAPK signaling pathway was once again present. Another proliferation‐related pathway present in the top 15 pathway list is the disease of signal transduction by growth factor receptors and second messengers. This pathway constituted 19 upregulated and 15 downregulated genes, amongst which were *DKK1* and *DKK4*, which were the two most upregulated genes in the G3 tumor. The network physical protein associations with G3 tumor DEGs showed a higher level of interactions than in G2 and G1 tumor networks, as 665 interactions between 247 proteins were found with a PPI enrichment *P*‐value of 1 × 10^−16^ (Fig. S5C).

### 3.6 Comparison of PanNET cell compartments between different tumor grades

**Fig. 9:**
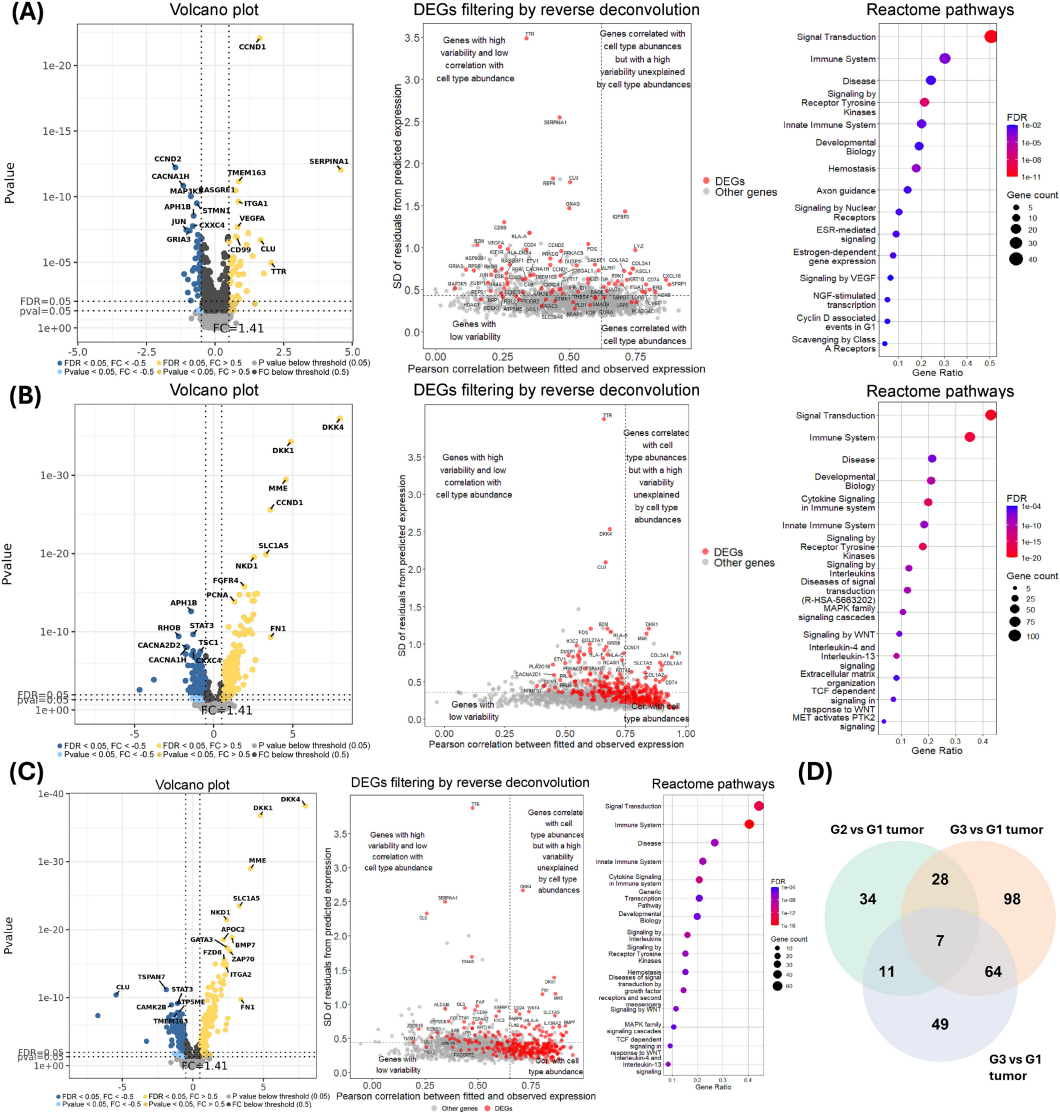
Comparison of tumor cell areas of illumination (AOIs) between different grades of the tumor. (A–C) Figures contain (1) volcano plots depicting the Log_2_ fold changes (*x*‐axis) and *P*‐values (*y*‐axis) of the assessed genes. (2) Scatterplots (DEGs filtering by reverse deconvolution) that display the results from Reverse Deconvolution where the *x*‐axis represents Pearson correlation between observed and fitted expression (based on cell abundance) and the *x*‐axis represents the standard deviation of Log_2_‐transformed residuals. Vertical and horizontal lines show third quartile (Q3) values used as cut‐offs. Any genes with correlation scores above Q3 and residual standard deviation values below Q3 were excluded from further STRING enrichment analysis. (3) Dot plots (Reactome pathways) of the top 15 Reactome pathways according to false discovery rate (FDR) value. The terms are sorted by gene count related to the term. (A) 24 grade 2 (G2) vs. 15 grade 1 (G1) tumor AOIs; (B) 6 grade 3 (G3) vs. 15 G1 tumor AOIs; (C) 6 G3 vs. 24 G2 tumor AOIs. (D) Venn diagram depicting the overlapping DEGs between the three comparisons.

Our final goal was to estimate the differences in gene expression in AOIs targeting tumor cells across the three PanNET grades. Similarly, as in the comparison of α‐SMA+ AOIs across grades, reverse deconvolution was also employed here to understand whether the variability identified in differentially expressed genes (DEGs) is significantly affected by the abundances of cell types (Table S14). There are noticeable differences in cell proportions between tumor AOIs across samples, particularly with infiltrated immune cells and intermixed stromal cells (see Fig. 1A). By comparing the tumor AOIs of G2 tumors against G1, we identified 83 DEGs (43 upregulated, 40 downregulated). Of these, three DEGs showed associations with cell type abundances and were removed from downstream analysis (Fig. 9A). In the list of 80 remaining DEGs (41 upregulated, 39 downregulated), 40 Reactome pathways were overrepresented (Table S15). The top 15 pathways (Fig. 9A) corresponded to immune system events, developmental biology (axon guidance, more specifically), cell proliferation, and estrogen‐mediated signaling. Another interesting observation here is the presence of signaling by the VEGF pathway, which involved two upregulated genes (*KDR*, *VEGFA*) and three downregulated genes (*NRP1*, *PRKACB*, *ROCK1*) in G2 tumor AOIs compared to G1. Amongst these 40 overrepresented pathways there were terms related to cell proliferation (Cyclin D‐associated events in G1, Mitotic G1 phase, and G1/S transition) and signaling by NTRK1 pathway, which included five downregulated genes found in G2 tumor AOIs compared to G1 (*ID2*, *ASCL1*, *FOS*, *ID1*, *SOS1*). NTRK1 regulates both cellular proliferation and neuronal differentiation according to the Reactome database. In the STRING network of physical protein associations, 36 physical interactions between 34 out of 83 proteins were identified at a PPI enrichment *P*‐value of 0.001 (Fig. S6A).

In both G3 vs. G1 and G3 vs. G2 comparisons, the amount of DEGs was substantially higher (Fig. 9D), 307 (161 upregulated, 146 downregulated) and 307 (161 upregulated, 146 downregulated); however, the proportions of genes related to the cell mixing were also much higher as shown by reverse deconvolution results (Fig. 9B,C). As a result, we reduced the list of DEGs from 307 to 197 (84 upregulated, 113 downregulated) in the G3 vs. G1 list and from 294 to 131 DEGs (58 upregulated, 73 downregulated) in the G3 vs. G2 list for further downstream analyses. As a result, 160 overrepresented pathways were identified in G3 vs. G1 and 141 in G3 vs. G2 tumor AOI comparisons (Table S15). Amongst the top 15 overrepresented pathways in the G3 vs. G1 scenario, there are a multitude of pathways related to cell proliferation (Diseases of signal transduction by growth factor receptors and second messengers, MAPK family signaling cascades, signaling by WNT, TCF‐dependent signaling in response to WNT) and one cell motility related signaling pathway (MET activates PTK2 signaling). A similar result can be observed for the G3 vs. G2 comparison as here, amidst the top 15 pathways, the MAPK and WNT signaling‐related pathways are also present. In both comparisons, the most pronounced genes in WNT signaling‐related pathways are *DKK4* and *DKK1*, as in both comparisons, these two genes were highly upregulated in G3 tumor (Fig. 9B,C). These genes exhibit a considerable correlation with cell type abundances. Still, their variability is not entirely explained by differences in cell abundances between samples, as indicated by their high residual standard deviations. PPI interaction networks for both G3 comparisons are depicted in Fig. S6B,C.

## 4 Discussion

In this study, using the DSP technology, we were able to segment the eight non‐functional PanNET tissues and evaluate the gene expression of 1800 cancer‐associated genes in three different AOIs: α‐SMA+ stroma, tumor adjacent to the α‐SMA+ stroma and tumor non‐adjacent to the α‐SMA+ stroma. As a result, we were able to identify gene expression profiles characteristic of α‐SMA+ stroma in PanNETs and assess whether tumor cells in regions enriched with α‐SMA+ stromal cells have different gene expression patterns compared to tumor cells in regions with poor α‐SMA+ stromal cell presence. Additionally, the DSP technology allowed us to identify and extract gene expression profiles specifically from pancreatic islets, which contain α, β, δ neuroendocrine cells. Since PanNETs are known to arise from either α or β cells of islets [41], comparing tumor AOIs to islet AOIs enabled us to more precisely examine the differences in gene expression profiles between PanNET cells and their neuroendocrine counterparts.

The expression levels of α‐SMA in the stromal compartment of the tumors have been linked to worse clinical outcomes and therapeutic resistance [17, 18, 42]. One of the principal explanations for such observations is the fact that α‐SMA is a marker for CAFs which arise from normal fibroblasts via TGF‐β, osteopontin (OPN), interleukin‐1β (IL‐1β), PDGF, TNF‐α, and FGF signals from the tumor [43, 44]. These signals, in turn, activate TGF‐β, PI3K/AKT, Ras/MAPK signaling pathways, and NF‐κB signaling pathways, leading to fibroblast transformation [44, 45]. Investigating these classical markers in our data, we did observe a statistically significant increase of *TNF* (TNF‐α) in G1 tumors, *FGF14* in G2 tumors, and *FGF8*, *FGF18*, and *PDGFA* in G3 tumor in comparison to pancreatic islet cells (Table S12). In addition, we found that PDGF receptor beta (*PDGFR*) expression was significantly higher within α‐SMA+ compartments in all three tumor grades than in other analyzed tissue compartments.

The G3 tumor also had overexpression of PDGF receptor alpha (*PDGFRA*). The pathway enrichment analysis showed that *PDGFRB and PDGFRA* were involved in Reactome pathway R‐HSA‐2219528 (PI3K/AKT Signaling in Cancer), which was overrepresented in DEGs found in the α‐SMA+ stroma of G2 and G3 tumors. The expression of PDGFRs has already been observed in stromal tissues of midgut NETs [46], and this data further adds a layer of evidence that the PDGF/PDFGR axis may also exist in PanNETs. Another interesting observation is regarding the *NOTCH3* receptor gene in α‐SMA+ stroma. There is rising evidence that cancer cells and fibroblasts interact directly through the cell surface ligand JAG1 and NOTCH receptors, and this, in turn, may also lead to fibroblast transformation into CAFs, driving the process of CAF‐mediated angiogenesis and extracellular matrix modification [47, 48]. A previous study in oral squamous cell carcinoma had shown that NOTCH3 is indeed overexpressed in CAFs, contributes to angiogenesis, and has been proposed as a therapeutic target [48]. Our data shows that the *NOTCH3* receptor gene is highly expressed in α‐SMA+ stroma compartments across all three tumor grades, and its ligand gene *JAG1* is upregulated in tumor compartments of G1 tumors. Considering the overexpression of NOTCH3 in α‐SMA+ stroma compartments, we can assume the possibility that the NOTCH axis may also be present in PanNET stroma.

Once fibroblasts are transformed and express α‐SMA, they can contribute to tumorigenesis in multiple ways. One of the main components that CAFs can regulate in TME is the extracellular matrix and immune infiltrates [32, 49]. One essential gene that plays a role in ECM modification is the stroma collagen gene [50]. Stroma‐derived collagens have been previously studied in terms of cancer progression, and in different combinations, they can either promote or inhibit tumorigenesis. For example, a study regarding PDAC showed that overexpression of type 1 collagen could promote tumorigenesis [51]. Another *in‐silico*‐based study that analyzed multiple publicly available datasets identified collagen genes *COL1A1*, *COL1A2*, *COL3A1*, and *COL5A1* as key CAF markers in gastric cancers [52]. These same collagen genes were also upregulated in α‐SMA+ stroma across all three tumor grades in our data. Besides the aforementioned collagen genes, we also observed upregulation of COL*5A2 and COL6A3* in G3 and G1 tumor/s in α‐SMA+ stroma. Another notable finding was gene *FN1*, which encodes yet another stromal CAF‐derived structural protein [52], and aside from being associated with invasiveness and tumor growth [52, 53], some studies have shown that its expression positively correlates with immune infiltrates [37, 39, 54]. In our data, we observe that the expression of *FN1* was significantly higher in α‐SMA+ stroma than in tumor or normal tissues across all three tumor grades. Its expression values also showed a moderate association with immune cell infiltrates within tumor AOIs (Fig. 5). Considering previous findings on *FN1* in other malignancies, it would be valuable to see future functional studies on fibronectin in PanNETs to evaluate its potential role as a regulator of cellular migration and impact on tumor cell proliferation.

Aside from identifying the gene profiles and associated pathways within the α‐SMA+ stroma of PanNETs, our other goal was to evaluate the impact of α‐SMA+ stroma on tumor cells' gene expression profiles. Since CAFs in the stroma and tumor cells communicate via cell‐to‐cell interactions and paracrine signals in both ways [55], we expected some differences in gene expression profiles of tumor cells immediately adjacent to α‐SMA+ stroma and tumor cells non‐adjacent to α‐SMA+ stroma. This hypothesis has already been tested in an *in‐vitro* study by *Wiechec et al*., where the authors found 13 DEGs in 2D cultures of tumor cells co‐cultured with CAFs in comparison to tumor cells cultured without CAFs and 81 DEGs in 3D cultures [56]. While in our data, the differences are very marginal (Fig. 7), we did identify two genes of interest: *MMP9* and *COL1A1*. These were the two most upregulated genes in G3 tumor compartments adjacent to α‐SMA+ stromal cells; however, these results should be interpreted carefully as only the *MMP9* had an FDR value of < 0.05. Nevertheless, these genes have been reported in a previous *in‐vitro* study of a similar concept. Within the study, the authors found that *COL1A1* and *MMP9* were also amongst the most upregulated genes in tumor cells that were co‐cultured with CAFs [56]. Other studies have shown that these genes are involved in metastasis development [54, 55, 56], with MMP9 having a particularly vital role as it can degrade ECM and is one of the most widely studied metastatic markers [57].

Lastly, we also looked at the gene expression profiles of the tumor itself. Since the employed method allows us to investigate the RNA expression within specific AOIs, we were able to compare the tumor compartments against pancreatic islets of Langerhans in adjacent normal tissue, which contain α and β cells, the deemed progenitors PanNETs [58]. Currently, there are only a few studies that have used either the traditional bulk RNA‐seq or microarray approach to investigate the PanNET tissues [22, 59, 60]. Moreover, there are only two studies that have included the islets as reference samples when comparing the gene expression profiles [61, 62]. When we compared each grade separately in our study, we first observed that five genes were consistently amongst the top 10 downregulated genes across all three tumor grades (Fig. 6E). These five genes have been linked to both pancreatic cancer and neuroendocrine tumors, particularly *CD99*, where the loss of it in PanNETs has previously been associated with a worse prognosis [63, 64, 65, 66, 67]. Furthermore, the *RBP4* was also found to be downregulated in the Dilley et al. study, where the authors also used islets as a reference [62]. However, it is worth mentioning that the overall overlapping DEG count with this study was relatively low as different gene panels were used, and authors analyzed Multiple Endocrine Neoplasia type 1 PanNETs. In contrast, we analyzed sporadic tumors [62].

Analyzing the pathways overrepresented in the list of dysregulated genes across all three tumor grades, we identified a multitude of cancer‐related pathways that have also been associated with neuroendocrine tumors [68]. Of these, the notable pathways were R‐HSA‐2219528 (PI3K/AKT Signaling in Cancer), which was overrepresented in DEGs of G1 and G3 tumors, and R‐HSA‐157118 (Signaling by NOTCH) in G2 and G3 tumors. Another finding was the overrepresentation of MAPK signaling pathways, particularly in G2 and G3 tumors. MAPK signaling is implicated to play a role in tumor development as it is activated by growth factor receptors, and the resulting signaling cascades lead to cellular proliferation and survival [69]. In neuroendocrine tumors, VEGF signaling pathways have also been given attention [70]. In our data, the VEGF pathways were overrepresented in G1 and G3 tumors; however, the expression of the pathways core gene *VEGFA* was lower in both G1 and G3 tumors compared to islets. VEGF is known to play an essential role in neuroendocrine tumor development by stimulating angiogenesis [21]. A study by Zhang et al. found that GEP‐NETs tumor cells express VEGFA in higher levels compared to stroma [71], which was also the case in our study (Fig. 5A); however, our study also shows that the expression levels of VEGF seem to be lower in PanNET cells than in islet cells. This result is not entirely unexpected as literature shows *VEGFA* is also critically important for islet cell development [72], and the comparison of VEGFA expression in islet cells and PanNET cells has not been studied yet. Lastly, signaling by WNT was also overrepresented in DEGs of G2 and G3 tumors. In G3 tumors regarding the WNT signaling pathways, we observed two genes that were two of the most upregulated DEGs (*DKK1* and *DKK4*). The Secreted Dickkopf (Dkk) proteins are known to be major elements in Wnt signaling, where they function as inhibitors of canonical Wnt signaling by blocking the interaction with LRP5/6 [73]. The role of these proteins within tumorigenesis remains unknown, as recent literature suggests that these Wnt antagonists can have both tumor‐suppressive and oncogenic effects [74]. Nevertheless, the overexpression of both DKK1 and DKK4 has been observed in pancreatic cancers and proposed as potential therapeutic targets [75, 76].

Overall, the results of PanNET and islet cell comparison further support the evidence that the classical PI3K/AKT, NOTCH, MAPK, and VEGF signaling pathways may play a role in PanNET development, and it would be interesting to see further functional studies on these pathways to develop novel treatment strategies.

After identifying the core gene expression profiles in α‐SMA+ stromal compartments and tumor compartments, we also carried out the comparison of both α‐SMA+ and tumor cell AOIs across different tumor grades to evaluate the changes in gene expression of both of these compartments as the tumor advances. There is a limited amount of knowledge regarding the changes in gene expression within PanNETs as, according to our knowledge, there is only one bulk RNA‐seq study by Simbolo et al. that carries out the comparison between grades of resected PanNET tissues [22]. In the study, it was discovered that the G3 vs. G1 tumor comparison had the highest amount of DEGs (2757), while G2 vs. G1 had the lowest (203). Based on these results, the authors concluded that G3 tumors are highly different from G1/G2 regarding gene expression profiles as they identified a total of 1104 overlapping DEGs between G3 vs. G1/G2 comparisons [22]. Here, we observe a similar result in our tumor cells containing AOI analysis data, as the lowest number of DEGs was identified in the G2 vs. G1 comparison (80), while the highest number of DEGs was in G3 vs. G1 comparison (197). As for the G3 vs. G2, we observed 131 DEGs for tumor compartments, which amounted to a total of 64 overlapping DEGs between G3 vs. G1/G2 comparisons, supporting the previous observation [22] that G3 tumors have their own unique gene expression profile that is different from G1/G2 tumors. A similar trend could not be observed concerning the stromal comparison by grades. While the comparisons with G3 tumor initially had the highest number of DEGs, most of the genes were highly correlated with cell type abundances as indicated by reverse deconvolution analysis. As a result, the highest amount of DEGs after filtering was observed for G2 vs. G1 comparison (51). However, these results are limited by the small sample size, making it difficult to draw any clear conclusions, as only one G3 tumor was analyzed.

This study has several limitations. As denoted previously, the main limitation of this study is that we analyzed only one retrospective case of G3 tumor; this can be attributed to the fact that in a clinical setting, G3 tumors are the rarest form of PanNETs. The second limitation was encountered during the spatial profiling preparation steps. Not all tumors were successfully stained with synaptophysin fluorescent antibody, even though all tumors were histopathologically confirmed to express synaptophysin. This issue could be attributed to the low sensitivity of the Synaptophysin antibody selected for GeoMx analysis. As a result, for samples PanNET1, PanNET3, and PanNET4, segmentation to acquire tumor and islet cell AOIs was performed based on synaptophysin immunofluorescence signal. A custom geometric‐based segmentation, which relied on tissue/cell morphology, was employed for the remaining samples. Despite this, additional PCA and heatmap analyses did not indicate a substantial unaccounted batch effect within the data introduced by the two different segmentation strategies. The third limitation is that the stromal component analysis was restricted to only α‐SMA‐expressing stromal cells. As such, it is impossible to clearly state the cellular composition and gene expression of tumor stroma that was α‐SMA‐negative. It would be beneficial to include additional stromal markers, especially CAF‐related markers such as FAP, PDGF receptors, and FSP‐1.

## 5 Conclusions

In summary, this study presents valuable information regarding PanNET biology, providing a cornerstone for future functional studies. Using the DSP approach, we were able to characterize PanNETs with a higher resolution as we analyzed α‐SMA+ stromal cells and tumor cells separately. This allowed us to identify potential core genes in PanNET stroma (*COL1A1*, *COL1A2*, *COL3A1*, *COL5A1*, *COL5A2*, *COL6A3*, and *FN1*) and tumor (*RBP4*, *SPINK1*, *PLA2G1B*, *EGR1*, and *CD99*), outlay the altered molecular pathways and pinpoint the potential mechanisms of crosstalk between tumor and stroma (*PDGF/PDGFR* axis, *NOTCH3*, *MMP9*). We also demonstrate that as PanNETs advance in grade, the gene expression profiles change within the tumor and, to some extent, in the stroma.

## Conflict of interest

The authors declare no conflict of interest.

## Author contributions

HN – carried out the digital spatial profiling analysis, bioinformatics analysis, and manuscript preparation; RP, RS, IM – performed additional statistical data analyses, revisions, and experiment planning; SV and JE – patient recruitment, clinical data collection, and manuscript preparation; AG, AP, NS – patient recruitment and clinical data collection; JN – additional histopathology analyses and manuscript revisions, AB, MGR – clinical data collection and tissue sample preparation; lastly, IR‐C – assisted with histopathology, ROI selection during the digital spatial profiling analysis, and manuscript revisions. VR – manuscript preparation, study design, and correspondence.

### Peer review

The peer review history for this article is available at https://www.webofscience.com/api/gateway/wos/peer‐review/10.1002/1878‐0261.13727.

## Supporting information


**Fig. S1.** Representative images of ATRX/DAXX, PDX1/ARX marker immunohistochemistry analysis.
**Fig. S2.** Clustering and principal component analysis of tumor and islet cell areas of illumination.
**Fig. S3.** Stacked bar chart of relative cell type abundances in tumor and alpha‐smooth muscle actin‐positive cell areas of illumination.
**Fig. S4.** STRING networks of physical protein associations depicting the physical interactions between proteins of differentially expressed genes from the comparison of alpha‐smooth muscle actin‐positive (α‐SMA+) areas of illumination between different tumor grades.
**Fig. S5.** STRING networks of physical protein associations depicting the physical interactions between proteins of differentially expressed genes from the comparison of tumor cell areas of illumination against pancreatic islet cell areas of illumination across all three tumor grades.
**Fig. S6.** STRING networks of physical protein associations depicting the physical interactions between proteins of differentially expressed genes from the comparison of tumor cell areas of illumination between different tumor grades.
**Table S1.** Results of immunohistochemistry analysis of ATRX/DAXX, PDX1/ARX markers.
**Table S2.** Raw counts of 1482 genes in all 104 areas of illumination analyzed in the study.
**Table S3.** Q3 normalized counts of 1482 genes in all 104 areas of illumination analyzed in the study.
**Table S4.** Metadata of all 104 analyzed areas of illumination.
**Table S5.** Spatial profiling of tumor tissue.
**Table S6.** Cell deconvolution matrices of all tumor and alpha‐smooth muscle actin‐expressing stromal cell areas of illumination.
**Table S7.** Results differential expression of alpha‐smooth muscle actin‐expressing stromal cell areas of illumination against the tumor, acinar compartment, and islet cell areas of illumination.
**Table S8.** Results of STRING functional enrichment analysis using overlapping differentially expressed genes as an input from Table S7.
**Table S9.** Alpha‐smooth muscle actin‐expressing stromal cell areas of illumination comparison across different tumor grades.
**Table S10.** Results of STRING functional enrichment analysis by using differentially expressed genes from alpha‐smooth muscle actin‐expressing stromal cell areas of illumination comparison across different tumor grades.
**Table S11.** Comparison of tumor areas of illumination from regions of interest with rich adjacent alpha‐smooth muscle actin‐expressing stromal cell presence vs. tumor areas of illumination with poor adjacent alpha‐smooth muscle actin‐expressing stromal cell presence.
**Table S12.** Comparison of tumor areas of illumination vs. pancreatic islet cell areas of illumination from non‐tumor tissue.
**Table S13.** Results of STRING functional enrichment analysis by using differentially expressed genes from the comparison of tumor areas of illumination vs. pancreatic islet cell areas of illumination from non‐tumor tissue.
**Table S14.** Tumor cell areas of illumination comparison across different tumor grades.
**Table S15.** Results of STRING functional enrichment analysis by using differentially expressed genes from tumor cell areas of illumination comparison across different tumor grades.

## Data Availability

All datasets containing raw/normalized counts and metadata information on each AOI analyzed in this study are described in the materials and methods section and are available in the supplementary material. Any additional datasets and information can be requested via the corresponding author/s.
