# Supplementary material for: Tumor and α‐SMA‐expressing stromal cells in pancreatic neuroendocrine tumors have a distinct RNA profile depending on tumor grade
Source: Mol Oncol. 2024 Sep 8;19(3):659–81. doi: 10.1002/1878-0261.13727 (PMC11887665; doi:10.1002/1878-0261.13727)
Supplement: Supplementary file 1 — Fig. S1. Representative images of ATRX/DAXX, PDX1/ARX marker immunohistochemistry analysis. Fig. S2. Clustering and principal component analysis of tumor and islet cell areas of illumination. Fig. S3. Stacked bar chart of relative cell type abundances in tumor and alpha‐smooth muscle actin‐positive cell areas of illumination. Fig. S4. STRING networks of physical protein associations depicting the physical interactions between proteins of differentially expressed genes from the comparison of alpha‐smooth muscle actin‐positive (α‐SMA+) areas of illumination between different tumor grades. Fig. S5. STRING networks of physical protein associations depicting the physical interactions between proteins of differentially expressed genes from the comparison of tumor cell areas of illumination against pancreatic islet cell areas of illumination across all three tumor grades. Fig. S6. STRING networks of physical protein associations depicting the physical interactions between proteins of differentially expressed genes from the comparison of tumor cell areas of illumination between different tumor grades. Table S1. Results of immunohistochemistry analysis of ATRX/DAXX, PDX1/ARX markers. Table S2. Raw counts of 1482 genes in all 104 areas of illumination analyzed in the study. Table S3. Q3 normalized counts of 1482 genes in all 104 areas of illumination analyzed in the study. Table S4. Metadata of all 104 analyzed areas of illumination. Table S5. Spatial profiling of tumor tissue. Table S6. Cell deconvolution matrices of all tumor and alpha‐smooth muscle actin‐expressing stromal cell areas of illumination. Table S7. Results differential expression of alpha‐smooth muscle actin‐expressing stromal cell areas of illumination against the tumor, acinar compartment, and islet cell areas of illumination. Table S8. Results of STRING functional enrichment analysis using overlapping differentially expressed genes as an input from Table S7. Table S9. Alpha‐smooth muscle actin‐expressing str [file MOL2-19-659-s001.zip › Legends.docx]

## Supporting information

### Figures

**Figure S1.** - Representative images of ATRX/DAXX, PDX1/ARX marker immunohistochemistry analysis.

**Figure S2.** - Clustering and principal component analysis of tumor and islet cell areas of illumination.

**Figure S3.** - Stacked bar chart of relative cell type abundances in tumor and alpha-smooth muscle actin positive cell areas of illumination.

**Figure S4.** - STRING networks of physical protein associations depicting physical interactions between proteins of differentially expressed genes from the comparison of alpha-smooth muscle actin expressing areas of illumination between different tumor grades.

**Figure S5.** *-* STRING networks of physical protein associations depicting physical interactions between proteins of differentially expressed genes from the comparison of tumor cell areas of illumination against pancreatic islet cell areas of illumination across all three tumor grades.

**Figure S6.** - STRING networks of physical protein associations depicting physical interactions between proteins of differentially expressed genes from the comparison of tumor cell areas of illumination between different tumor grades

### Tables

**Table S1.** - Table S1. Results of immunohistochemistry analysis of ATRX/DAXX, PDX1/ARX markers.

**Table S2.** - Raw counts of 1482 genes in all 104 areas of illumination analyzed in the study.

**Table S3.** - Q3 normalized counts of 1482 genes in all 104 areas of illumination analyzed in the study.

**Table S4.** - Metadata of all 104 analyzed areas of illumination.

**Table S5.** - Spatial profiling of tumor tissue.

**Table S6.** - Cell deconvolution matrices of all tumor and alpha-smooth muscle actin expressing stromal cell areas of illumination.

**Table S7.** - Results differential expression of alpha-smooth muscle actin-expressing stromal cell areas of illumination against the tumor, acinar compartment, and islet cell areas of illumination.

**Table S8.** Results of STRING functional enrichment analysis using overlapping differentially expressed genes as an input from Table S7.

**Table S9.** Alpha-smooth muscle actin expressing stromal cell areas of illumination comparison across different tumor grades.

**Table S10.** Results of STRING functional enrichment analysis by using differentially expressed genes from alpha-smooth muscle actin expressing stromal cell areas of illumination comparison across different tumor grades.

**Table S11.** - Comparison of tumor areas of illumination from regions of interest with rich adjacent alpha-smooth muscle actin expressing stromal cell presence vs. tumor areas of illumination with poor adjacent alpha-smooth muscle actin expressing stromal cell presence

**Table S12.** Comparison of tumor areas of illumination vs. pancreatic islet cell areas of illumination from non-tumor tissue.

**Table S13.** Results of STRING functional enrichment analysis by using differentially expressed genes from the comparison of tumor areas of illumination vs. pancreatic islet cell areas of illumination from non-tumor tissue.

**Table S14.** Tumor cell areas of illumination comparison across different tumor grades.

**Table S15.** Results of STRING functional enrichment analysis by using differentially expressed genes from tumor cell areas of illumination comparison across different tumor grades.
