# Supplementary figures and images for: Tumor and α‐SMA‐expressing stromal cells in pancreatic neuroendocrine tumors have a distinct RNA profile depending on tumor grade
Source: Mol Oncol. 2024 Sep 8;19(3):659–81. doi: 10.1002/1878-0261.13727 (PMC11887665; doi:10.1002/1878-0261.13727)

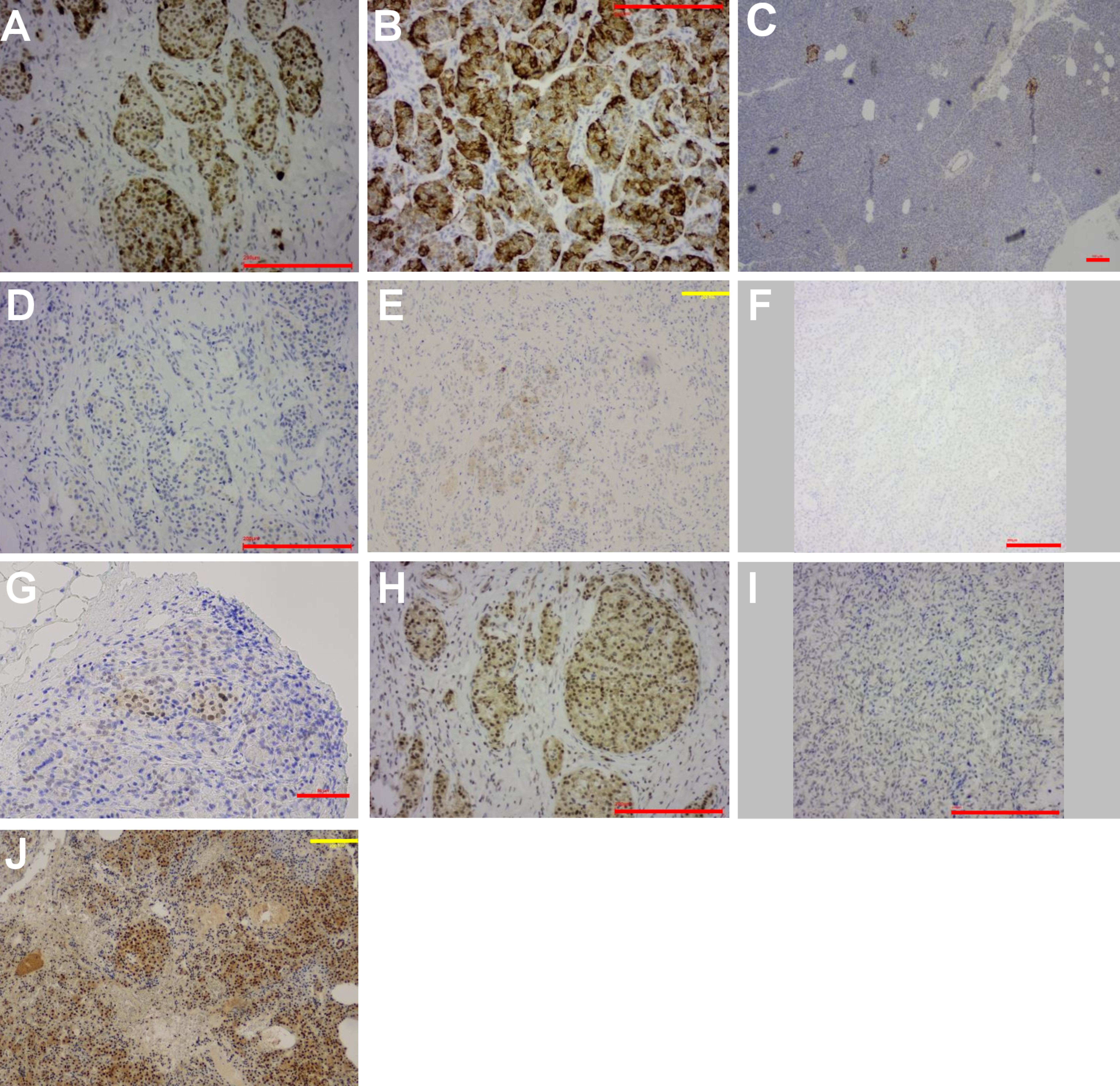

Supplement: Supplementary file 1 — Fig. S1. Representative images of ATRX/DAXX, PDX1/ARX marker immunohistochemistry analysis. Fig. S2. Clustering and principal component analysis of tumor and islet cell areas of illumination. Fig. S3. Stacked bar chart of relative cell type abundances in tumor and alpha‐smooth muscle actin‐positive cell areas of illumination. Fig. S4. STRING networks of physical protein associations depicting the physical interactions between proteins of differentially expressed genes from the comparison of alpha‐smooth muscle actin‐positive (α‐SMA+) areas of illumination between different tumor grades. Fig. S5. STRING networks of physical protein associations depicting the physical interactions between proteins of differentially expressed genes from the comparison of tumor cell areas of illumination against pancreatic islet cell areas of illumination across all three tumor grades. Fig. S6. STRING networks of physical protein associations depicting the physical interactions between proteins of differentially expressed genes from the comparison of tumor cell areas of illumination between different tumor grades. Table S1. Results of immunohistochemistry analysis of ATRX/DAXX, PDX1/ARX markers. Table S2. Raw counts of 1482 genes in all 104 areas of illumination analyzed in the study. Table S3. Q3 normalized counts of 1482 genes in all 104 areas of illumination analyzed in the study. Table S4. Metadata of all 104 analyzed areas of illumination. Table S5. Spatial profiling of tumor tissue. Table S6. Cell deconvolution matrices of all tumor and alpha‐smooth muscle actin‐expressing stromal cell areas of illumination. Table S7. Results differential expression of alpha‐smooth muscle actin‐expressing stromal cell areas of illumination against the tumor, acinar compartment, and islet cell areas of illumination. Table S8. Results of STRING functional enrichment analysis using overlapping differentially expressed genes as an input from Table S7. Table S9. Alpha‐smooth muscle actin‐expressing str [file MOL2-19-659-s001.zip › mol213727-sup-0001-FigureS1.tiff]

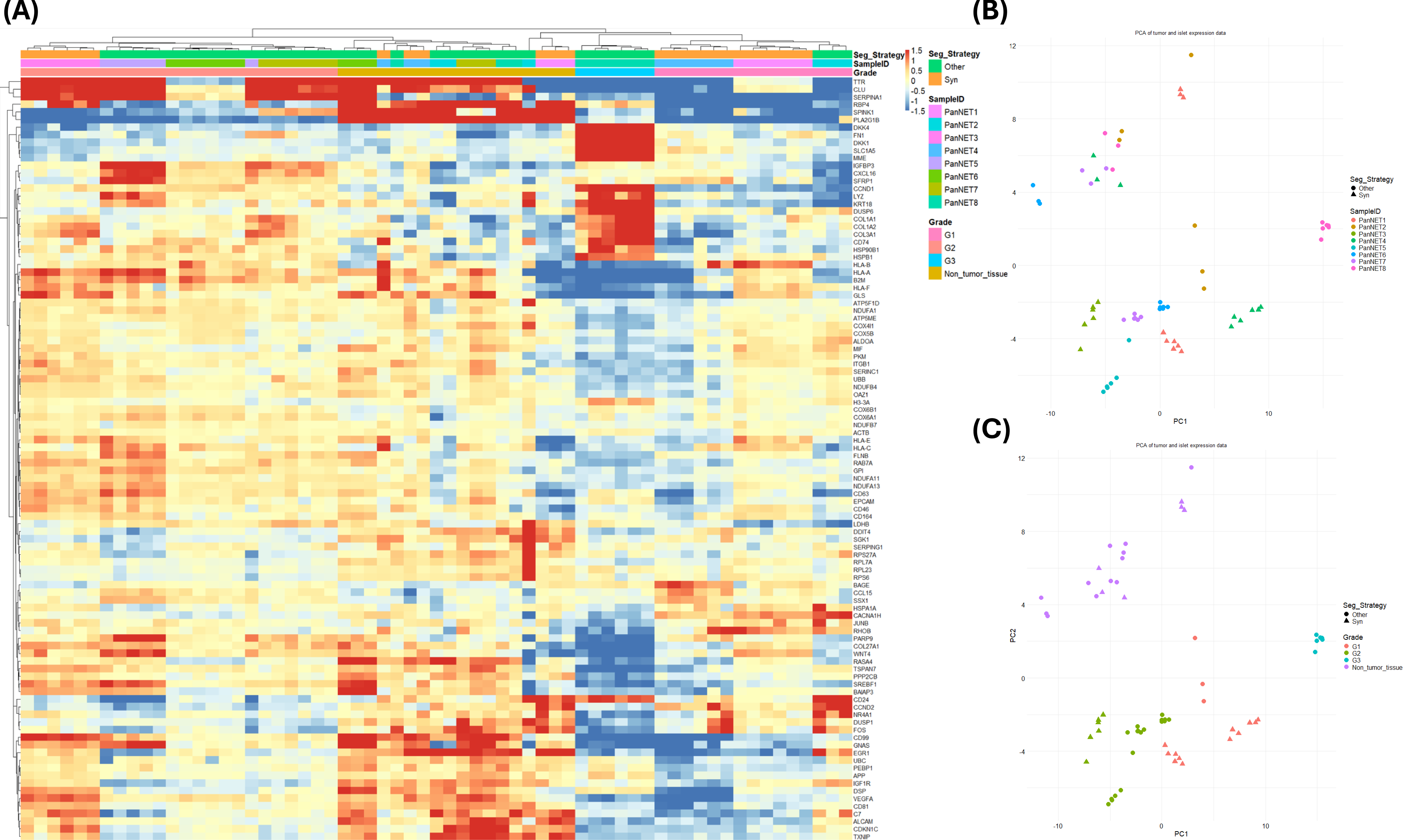

Supplement: Supplementary file 1 — Fig. S1. Representative images of ATRX/DAXX, PDX1/ARX marker immunohistochemistry analysis. Fig. S2. Clustering and principal component analysis of tumor and islet cell areas of illumination. Fig. S3. Stacked bar chart of relative cell type abundances in tumor and alpha‐smooth muscle actin‐positive cell areas of illumination. Fig. S4. STRING networks of physical protein associations depicting the physical interactions between proteins of differentially expressed genes from the comparison of alpha‐smooth muscle actin‐positive (α‐SMA+) areas of illumination between different tumor grades. Fig. S5. STRING networks of physical protein associations depicting the physical interactions between proteins of differentially expressed genes from the comparison of tumor cell areas of illumination against pancreatic islet cell areas of illumination across all three tumor grades. Fig. S6. STRING networks of physical protein associations depicting the physical interactions between proteins of differentially expressed genes from the comparison of tumor cell areas of illumination between different tumor grades. Table S1. Results of immunohistochemistry analysis of ATRX/DAXX, PDX1/ARX markers. Table S2. Raw counts of 1482 genes in all 104 areas of illumination analyzed in the study. Table S3. Q3 normalized counts of 1482 genes in all 104 areas of illumination analyzed in the study. Table S4. Metadata of all 104 analyzed areas of illumination. Table S5. Spatial profiling of tumor tissue. Table S6. Cell deconvolution matrices of all tumor and alpha‐smooth muscle actin‐expressing stromal cell areas of illumination. Table S7. Results differential expression of alpha‐smooth muscle actin‐expressing stromal cell areas of illumination against the tumor, acinar compartment, and islet cell areas of illumination. Table S8. Results of STRING functional enrichment analysis using overlapping differentially expressed genes as an input from Table S7. Table S9. Alpha‐smooth muscle actin‐expressing str [file MOL2-19-659-s001.zip › mol213727-sup-0002-FigureS2.tif]

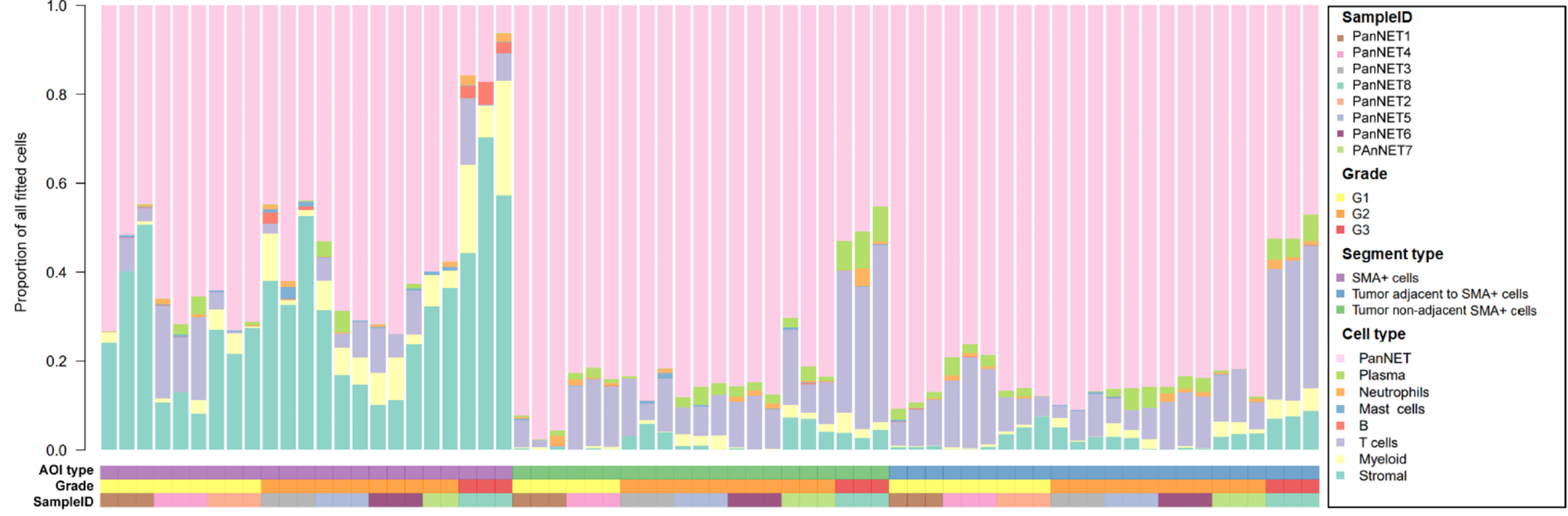

Supplement: Supplementary file 1 — Fig. S1. Representative images of ATRX/DAXX, PDX1/ARX marker immunohistochemistry analysis. Fig. S2. Clustering and principal component analysis of tumor and islet cell areas of illumination. Fig. S3. Stacked bar chart of relative cell type abundances in tumor and alpha‐smooth muscle actin‐positive cell areas of illumination. Fig. S4. STRING networks of physical protein associations depicting the physical interactions between proteins of differentially expressed genes from the comparison of alpha‐smooth muscle actin‐positive (α‐SMA+) areas of illumination between different tumor grades. Fig. S5. STRING networks of physical protein associations depicting the physical interactions between proteins of differentially expressed genes from the comparison of tumor cell areas of illumination against pancreatic islet cell areas of illumination across all three tumor grades. Fig. S6. STRING networks of physical protein associations depicting the physical interactions between proteins of differentially expressed genes from the comparison of tumor cell areas of illumination between different tumor grades. Table S1. Results of immunohistochemistry analysis of ATRX/DAXX, PDX1/ARX markers. Table S2. Raw counts of 1482 genes in all 104 areas of illumination analyzed in the study. Table S3. Q3 normalized counts of 1482 genes in all 104 areas of illumination analyzed in the study. Table S4. Metadata of all 104 analyzed areas of illumination. Table S5. Spatial profiling of tumor tissue. Table S6. Cell deconvolution matrices of all tumor and alpha‐smooth muscle actin‐expressing stromal cell areas of illumination. Table S7. Results differential expression of alpha‐smooth muscle actin‐expressing stromal cell areas of illumination against the tumor, acinar compartment, and islet cell areas of illumination. Table S8. Results of STRING functional enrichment analysis using overlapping differentially expressed genes as an input from Table S7. Table S9. Alpha‐smooth muscle actin‐expressing str [file MOL2-19-659-s001.zip › mol213727-sup-0003-FigureS3.tif]

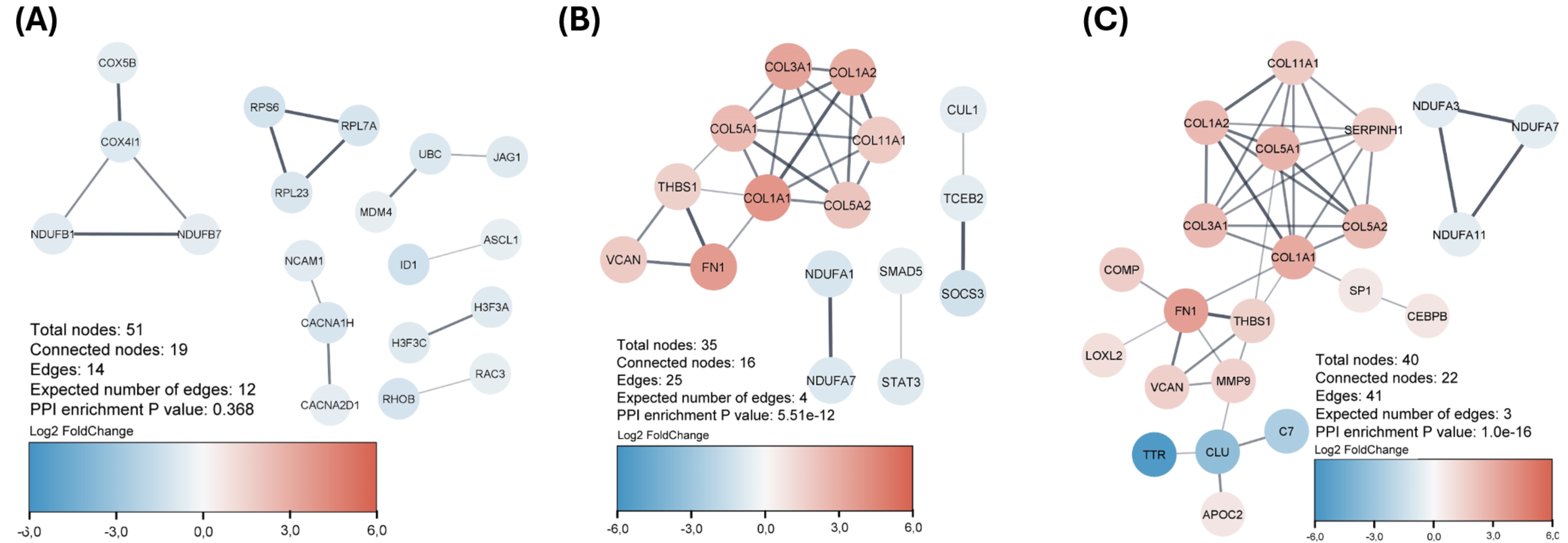

Supplement: Supplementary file 1 — Fig. S1. Representative images of ATRX/DAXX, PDX1/ARX marker immunohistochemistry analysis. Fig. S2. Clustering and principal component analysis of tumor and islet cell areas of illumination. Fig. S3. Stacked bar chart of relative cell type abundances in tumor and alpha‐smooth muscle actin‐positive cell areas of illumination. Fig. S4. STRING networks of physical protein associations depicting the physical interactions between proteins of differentially expressed genes from the comparison of alpha‐smooth muscle actin‐positive (α‐SMA+) areas of illumination between different tumor grades. Fig. S5. STRING networks of physical protein associations depicting the physical interactions between proteins of differentially expressed genes from the comparison of tumor cell areas of illumination against pancreatic islet cell areas of illumination across all three tumor grades. Fig. S6. STRING networks of physical protein associations depicting the physical interactions between proteins of differentially expressed genes from the comparison of tumor cell areas of illumination between different tumor grades. Table S1. Results of immunohistochemistry analysis of ATRX/DAXX, PDX1/ARX markers. Table S2. Raw counts of 1482 genes in all 104 areas of illumination analyzed in the study. Table S3. Q3 normalized counts of 1482 genes in all 104 areas of illumination analyzed in the study. Table S4. Metadata of all 104 analyzed areas of illumination. Table S5. Spatial profiling of tumor tissue. Table S6. Cell deconvolution matrices of all tumor and alpha‐smooth muscle actin‐expressing stromal cell areas of illumination. Table S7. Results differential expression of alpha‐smooth muscle actin‐expressing stromal cell areas of illumination against the tumor, acinar compartment, and islet cell areas of illumination. Table S8. Results of STRING functional enrichment analysis using overlapping differentially expressed genes as an input from Table S7. Table S9. Alpha‐smooth muscle actin‐expressing str [file MOL2-19-659-s001.zip › mol213727-sup-0004-FigureS4.tif]

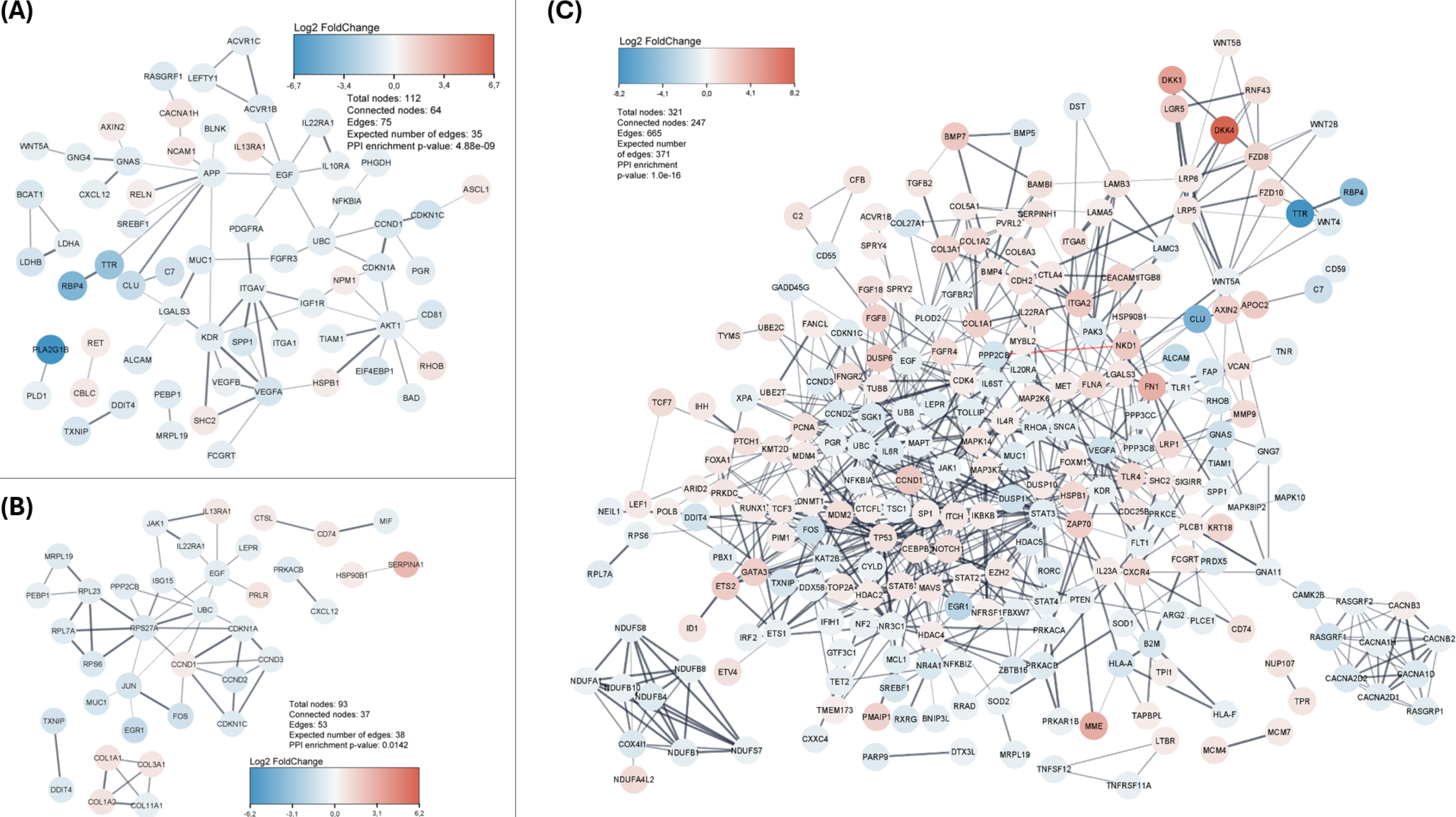

Supplement: Supplementary file 1 — Fig. S1. Representative images of ATRX/DAXX, PDX1/ARX marker immunohistochemistry analysis. Fig. S2. Clustering and principal component analysis of tumor and islet cell areas of illumination. Fig. S3. Stacked bar chart of relative cell type abundances in tumor and alpha‐smooth muscle actin‐positive cell areas of illumination. Fig. S4. STRING networks of physical protein associations depicting the physical interactions between proteins of differentially expressed genes from the comparison of alpha‐smooth muscle actin‐positive (α‐SMA+) areas of illumination between different tumor grades. Fig. S5. STRING networks of physical protein associations depicting the physical interactions between proteins of differentially expressed genes from the comparison of tumor cell areas of illumination against pancreatic islet cell areas of illumination across all three tumor grades. Fig. S6. STRING networks of physical protein associations depicting the physical interactions between proteins of differentially expressed genes from the comparison of tumor cell areas of illumination between different tumor grades. Table S1. Results of immunohistochemistry analysis of ATRX/DAXX, PDX1/ARX markers. Table S2. Raw counts of 1482 genes in all 104 areas of illumination analyzed in the study. Table S3. Q3 normalized counts of 1482 genes in all 104 areas of illumination analyzed in the study. Table S4. Metadata of all 104 analyzed areas of illumination. Table S5. Spatial profiling of tumor tissue. Table S6. Cell deconvolution matrices of all tumor and alpha‐smooth muscle actin‐expressing stromal cell areas of illumination. Table S7. Results differential expression of alpha‐smooth muscle actin‐expressing stromal cell areas of illumination against the tumor, acinar compartment, and islet cell areas of illumination. Table S8. Results of STRING functional enrichment analysis using overlapping differentially expressed genes as an input from Table S7. Table S9. Alpha‐smooth muscle actin‐expressing str [file MOL2-19-659-s001.zip › mol213727-sup-0005-FigureS5.tif]

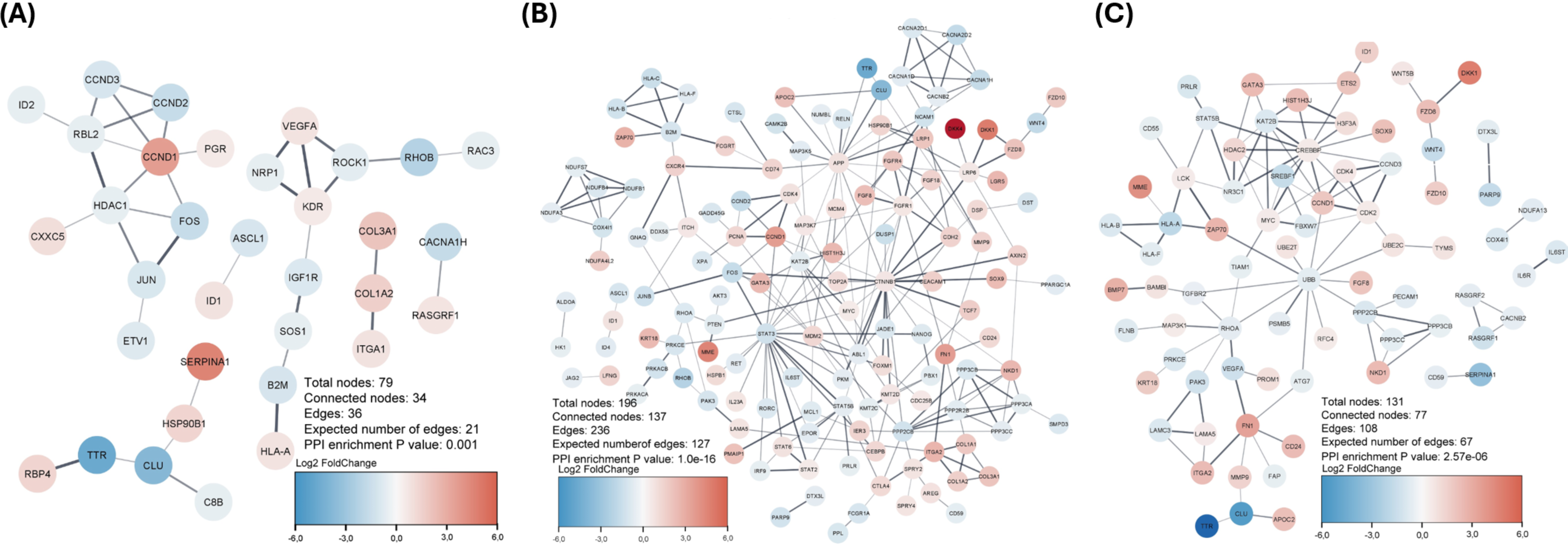

Supplement: Supplementary file 1 — Fig. S1. Representative images of ATRX/DAXX, PDX1/ARX marker immunohistochemistry analysis. Fig. S2. Clustering and principal component analysis of tumor and islet cell areas of illumination. Fig. S3. Stacked bar chart of relative cell type abundances in tumor and alpha‐smooth muscle actin‐positive cell areas of illumination. Fig. S4. STRING networks of physical protein associations depicting the physical interactions between proteins of differentially expressed genes from the comparison of alpha‐smooth muscle actin‐positive (α‐SMA+) areas of illumination between different tumor grades. Fig. S5. STRING networks of physical protein associations depicting the physical interactions between proteins of differentially expressed genes from the comparison of tumor cell areas of illumination against pancreatic islet cell areas of illumination across all three tumor grades. Fig. S6. STRING networks of physical protein associations depicting the physical interactions between proteins of differentially expressed genes from the comparison of tumor cell areas of illumination between different tumor grades. Table S1. Results of immunohistochemistry analysis of ATRX/DAXX, PDX1/ARX markers. Table S2. Raw counts of 1482 genes in all 104 areas of illumination analyzed in the study. Table S3. Q3 normalized counts of 1482 genes in all 104 areas of illumination analyzed in the study. Table S4. Metadata of all 104 analyzed areas of illumination. Table S5. Spatial profiling of tumor tissue. Table S6. Cell deconvolution matrices of all tumor and alpha‐smooth muscle actin‐expressing stromal cell areas of illumination. Table S7. Results differential expression of alpha‐smooth muscle actin‐expressing stromal cell areas of illumination against the tumor, acinar compartment, and islet cell areas of illumination. Table S8. Results of STRING functional enrichment analysis using overlapping differentially expressed genes as an input from Table S7. Table S9. Alpha‐smooth muscle actin‐expressing str [file MOL2-19-659-s001.zip › mol213727-sup-0006-FigureS6.tif]
